# Multi-task learning for predicting quality-of-life and independence in activities of daily living after stroke: a proof-of-concept study

**DOI:** 10.3389/fneur.2024.1449234

**Published:** 2024-09-27

**Authors:** Thi Nguyet Que Nguyen, Alejandro García-Rudolph, Joan Saurí, John D. Kelleher

**Affiliations:** ^1^Research Hub 4 - Digital Futures Research Hub, Technological University Dublin, Dublin, Ireland; ^2^Artificial Intelligence in Digital Health and Medicine (AIDHM), Technological University Dublin, Dublin, Ireland; ^3^Department of Research and Innovation, Institut Guttmann, Institut Universitari de Neurorehabilitació adscrit ala UAB, Barcelona, Spain; ^4^Departament de Medicina, Universitat Autonoma De Barcelona, Bellaterra, Spain; ^5^Fundació Institut d'Investigació en Ciències de la Salut Germans Trias i Pujol, Barcelona, Spain; ^6^School of Computer Science and Statistics, Trinity College Dublin, ADAPT Research Centre, Dublin, Ireland

**Keywords:** multi-task learning, task grouping, stroke, activities of daily living, quality-of-life, Barthel index, EQ-5D-3L

## Abstract

A health-related (HR) profile is a set of multiple health-related items recording the status of the patient at different follow-up times post-stroke. In order to support clinicians in designing rehabilitation treatment programs, we propose a novel multi-task learning (MTL) strategy for predicting post-stroke patient HR profiles. The HR profile in this study is measured by the Barthel index (BI) assessment or by the EQ-5D-3L questionnaire. Three datasets are used in this work and for each dataset six neural network architectures are developed and tested. Results indicate that an MTL architecture combining a pre-trained network for all tasks with a concatenation strategy conditioned by a task grouping method is a promising approach for predicting the HR profile of a patient with stroke at different phases of the patient journey. These models obtained a mean F1-score of 0.434 (standard deviation 0.022, confidence interval at 95% [0.428, 0.44]) calculated across all the items when predicting BI at 3 months after stroke (MaS), 0.388 (standard deviation 0.029, confidence interval at 95% [0.38, 0.397]) when predicting EQ-5D-3L at 6MaS, and 0.462 (standard deviation 0.029, confidence interval at 95% [0.454, 0.47]) when predicting the EQ-5D-3L at 18MaS. Furthermore, our MTL architecture outperforms the reference single-task learning models and the classic MTL of all tasks in 8 out of 10 tasks when predicting BI at 3MaS and has better prediction performance than the reference models on all tasks when predicting EQ-5D-3L at 6 and 18MaS. The models we present in this paper are the first models to predict the components of the BI or the EQ-5D-3L, and our results demonstrate the potential benefits of using MTL in a health context to predict patient profiles.

## 1 Introduction

Many medical instruments that are widely used to measure and assess the health status of a patient are structured questionnaires (1–3). To help the clinician improve treatment decisions, machine learning (ML) is often used to forecast patients' future conditions by predicting the answers of such questionnaires. To date, research on this type has focused on using conventional single-task learning (STL). In STL, a separate model is trained to predict either a value representing the summary of these answers (4, 5), or each element of a questionnaire independently (e.g. the study of Devlin et al. in (6)).

An understudied alternative to STL is to use multi-task learning (MTL) where a model is trained to make predictions for groups of related items in parallel, thereby allowing the model to learn to predict coherent sets of values across the items (7). In this study, we explore the benefit of MTL to predict the answers of structured questionnaires and compare our results to conventional STL models.

Health-related quality-of-life is a recognized tool for evaluating and assessing the health state of patients during post-acute stroke treatment (8). By the definition of the World Health Organization, quality-of-life (QoL) is “an individual's perception of their position in life in the context of the culture and value systems in which they live and in relation to their goals, expectations, standards and concerns” (9). However, the definition of QoL is still vague and ambiguous when used in different fields (10). In the health context, the concept of QoL can be defined as (i) a set of health-related (HR) outcomes or (ii) an outcome score that describes a patient's overall wellbeing or health (11).

The first QoL definition represents a broader conceptualization of QoL where QoL is composed of a set of HR outcomes describing and evaluating the ability of the patient in different physical activities of daily living (ADLs), as well as the psychological or cognitive aspects of the wellbeing of the patient. Such conceptualization can be measured using various questionnaires or surveys of patients that are administered during follow-up care, such as the stroke specific quality-of-life (SS-QoL) (12), EQ-5D (13, 14), or SF-36 (15). The answers of these surveys form a set of HR outcomes of the patients. For the second QoL definition, the outcome score used may be calculated by a similar measure of QoL impairment as mentioned earlier (e.g., EQ-5D, SS-QoL, SF-36). For each of these cases, the patient assessment is composed of multiple items. The overall outcome score of the assessment is either the sum of all the individual scores from each item (e.g., SS-QoL total score, SF-36 total score (16)), or a value that summarizes the item scores (e.g., EQ-5D index value (17)).

During stroke treatment and rehabilitation, healthcare professionals use instruments such as the Barthel index (BI) (18) or the functional independence measure (FIM) (19) to assess and track how patients are recovering with respect to different physical ADLs. ADL dependency is a common consequence post-stroke and persists in 35% of stroke survivors during the first year after stroke (20). ADL evaluation provides evidence for decision-making in treatment, rehabilitation, nursing and ultimately social participation after discharge (21). Similar to the concept of QoL, a patient's independence in ADL (iADL) can be assessed using either the set of answers of the questionnaire or the outcome score of the iADL assessment (e.g., the total score of BI or FIM).

However, the outcome scores of QoL and iADL assessments mask all the answers of the QoL and iADL questionnaires. Consequently, predicting the set of HR outcomes at a given time point after stroke may help clinicians to identify and prioritize the areas of need of the patient, thus improving the treatment for the patient to restore their functional abilities and QoL after stroke (22, 23). Inspired by previous studies (24, 25) on defining the QoL profile of the patient, in this work we treated the assessments of QoL and iADL, i.e., the EQ-5D-3L and BI respectively, as representing personalized HR profiles of patients.

To improve the care of patients during post-acute stroke treatment, we are interested in predicting the HR profile of the patient at a given time point after stroke. Especially, we are interested in predicting all the items of the BI and EQ-5D-3L assessments since several studies have shown their mutual support in the diagnosis and assessment of patients with stroke (26, 27). A set of multiple outcomes is a more complex structure than an outcome score, making the prediction of an HR profile a challenge. Given the inter-related multi-element structure of these instruments, a relevant and novel approach to predicting all the items of the HR profile is to use MTL to bridge the gap of applying predictive modeling for post-stroke rehabilitation.

MTL technique involves training an ML model to predict outcomes for multiple problems at the same time using a shared representation (28). MTL is different from the more standard ML approach of STL where a separate model is independently trained for each problem. Each problem can have two labels (binary classification), more than two labels (multinomial classification), or have a continuous numeric target (regression) (29). In recent years, neural-network-based MTL has demonstrated its advantages in different domains such as computer vision, speech recognition, natural language processing, multimedia data processing, reinforcement learning, and multi-modal problems (7, 30, 31).

The idea underpinning MTL is that it is sometimes possible to improve the prediction performance of a system across a set of related tasks by integrating the shared information across these tasks during the training of the system. This is generally achieved by training a system on all the tasks in parallel and sharing model parameters across the tasks. For example, in the case of a multi-task neural network the sharing of parameters can be achieved by sharing one or more layers of the network across multiple tasks. In this way, the learning signals from multiple tasks are integrated during the updates of these shared parameters (7). However, the benefit of using MTL is often dependent on the strength of the relationship between the sub-tasks that are learnt in parallel (32, 33). If MTL is applied to a set of tasks that are not related this can result in a more complex model with no benefit in prediction accuracy on any task.

Another potential problem with applying MTL is the so-called “Robin Hood effect”. The Robin Hood effect describes a situation in MTL where learning multiple tasks in parallel improves the predictive performance of one or more of the tasks but this gain is at the cost of reducing the predictive accuracy on some of the other tasks (34). Consequently, an important aspect of applying MTL is choosing the sets of tasks that are grouped and learned in parallel. A number of methods have been proposed to identify groups of tasks that will benefit from MTL, for example the grouping of tasks can be selected by using statistical calculation (e.g., dependency coefficients (35, 36)), by per-task loss estimation (e.g., higher order approximation strategy (37)) or by the calculation of the affinity between tasks (e.g., task-affinity grouping methodology (38)). Importantly, these grouping criteria may not agree with respect to what tasks to group. Thus, the selection of the criteria applied to define the task groups can be understood as a hyper-parameter of the MTL approach that can affect the overall effectiveness of the MTL system.

In this paper, we frame post-stroke HR profile prediction as a multi-task problem where the prediction of each item of an HR profile is treated as a learning task. We use STL, where a separate independent prediction model is trained for each task, as the baseline comparator. We investigate the efficacy of two different methods (task-affinity grouping (38) and Cramer's V task-association grouping) for identifying related tasks that the system may benefit from learning together, and propose and evaluate a number of strategies for constructing an MTL system. We also analyse the presence of the “Robin Hood effect” when comparing the performance between the novel proposed MTL against the baseline models such as STL or the classic MTL approach where all tasks are learned in parallel (where no task grouping is applied).

To the best of our knowledge, this research is the first time neural network-based MTL has been applied to the prediction of the details of the HR profile of patients with stroke, i.e., predicting the score of each BI item or each EQ-5D-3L items in parallel.

This paper is structured as follows. A review of the application of MTL in healthcare, with a particular focus on its use for stroke treatment in predicting BI and EQ-5D-3L assessment, is provided in Section 2. We then describe, in Section 3, the three studied clinical datasets. Section 4 describes the data handling methods we use in our experiments, and Section 5 details the ML architectures we study and develop in our experiments. Following that, Section 6 describes our experiments and the results obtained from each ML approach, as well as the comparison between the studied models. In Section 7, we discuss the advantages of the proposed MTL approach when comparing it to the STL and the classic MTL baseline models. Finally, we set out our conclusions and our perspective on future work in Section 8.

## 2 Related works: multi-task learning for the prediction of health-related outcomes

MTL has shown its potential in previous healthcare applications for predicting future outcomes at multiple HR dimensions of the patients (7, 30, 31), which helps to inform the clinician of the possible future states or risks that the patient could have. A recent example of MTL being used in a healthcare setting is Roy et al. (39) who introduced sequential subnetwork routing which is a sequential MTL deep recurrent neural network architecture that use electronic health records to predict the onset of multiple endpoints. These endpoints include the onset of specific organ dysfunctions and general clinical outcomes such as acute kidney injury, continuous renal replacement therapy, mechanical ventilation, vasoactive medications, mortality, and length of stay of the patient. Harutyunyan et al. (40) proposed an MTL long short-term memory-based network that trains on a multivariate clinical time series database to predict four targets in parallel: the risk of in-hospital mortality, the length of stay, physiologic decompensation, and phenotype classification of the patients. In the context of supporting the treatment of atrial fibrillation, an MTL model (41) combining a U-net for segmentation with a fully connected network for classification was developed to perform atrial segmentation and pre- or post-ablation classification, using gadolinium-enhanced magnetic resonance images. MTL has also been applied in neurological disorders, such as Alzheimer's disease diagnosis. Liu et al. (42) proposed an MTL neural network multi-channel learning model that simultaneously predicts brain disease and estimates the clinical score, using magnetic resonance imaging data and demographic information of patients. However, none of these works have focused on post-stroke patients, nor on predicting the elements of a QoL or iADL structured questionnaire.

In stroke treatment, the standard STL approach has been used to develop models to predict post-stroke BI total scores and this line of research has reported promising results for improving the care of patients (43). For example, Lin et al. (4) demonstrated the relative effectiveness (compared with support vector machines) of logistic regression and random forest models for predicting the total scores of BI at discharge. In (44), an STL approach based on support vector machines was used to predict the motor, cognitive and total score of the FIM and the total BI score of a patient with stroke at discharge from a rehabilitation unit. Generally, these methods were developed for predicting the total score or the rank of total BI score (e.g., low, medium or high rank).

However, when the total score of BI is used, this summary score masks the information of the activities where a patient's ability is impaired. This can result in practitioners and researchers finding it difficult to interpret the clinical meaning of BI summary scores or changes in these scores. The prediction of the specific ADL outcomes after stroke rehabilitation is a helpful means for clinicians to improve the accuracy of prognoses, set attainable goals, reach shared decisions, personalize rehabilitation plans, and inform patients and relatives (45). A recent study (6) proposed a tool that predicts the level of 9 out of the 10 items in the BI at 3, 6, 12 months after stroke (MaS). They train a separate Random Forest model for each BI item and prediction time point (i.e., they use an STL modeling approach). However, in that study the prediction of each BI item was scaled down to an independent binary classification problem, i.e. fully functional vs. any level of dependency. By comparison, in this paper, we assess the benefit of neural network-based MTL (as opposed to STL) for predicting the future levels of BI items of the patient while preserving the multi-level characteristic of such items.

Besides the mentioned works on BI, we are also interested in studies of ML applications on the EQ-5D-3L questionnaire. EQ-5D-3L is one type of EQ-5D instrument that aims to measure the health status of the patient (46) (more details about EQ-5D-3L is presented in Section 3.2.1). The answers of the EQ-5D questionnaire are usually collected and combined to generate a health profile (or health state) of a patient. There are 243 possible health profiles for EQ-5D-3L. Each EQ-5D-3L health profile can be then summarized to one value, the so-called EQ-5D-3L index value (EQ-5D-3L index, quality-of-life weights or utilities) (17). This index is calculated using a weighted sum of the scored answers to the questionnaire, and the EQ-5D-3L index ranges from 0 (indicating death) to 1 (indicating full health) (17). The set of weights used for converting the EQ-5D-3L health profile to EQ-5D-3L index value is called a value set, note that the weighting applied for the conversion can vary between studies (17, 47).

In the literature, multiple studies have been developed for mapping other QoL assessments to the EQ-5D-3L index value (48) and also for predicting EQ-5D-3L index value. For example, Zrubka et al. (49) recently proposed a study for evaluating the performance of ML methods (such as extreme gradient boosting classification, extreme gradient boosting regression and ordinary least squares regression) in predicting the EQ-5D-3L index value of patients in various health conditions using demographic data, disease-related variables and patient-reported outcome information. A study from Barbosa et al. (50) has applied linear regression to predict the EQ-5D-3L index and the Short Form 6 Health Survey Instrument of the patients at four time-points such as in the first seven days after stroke (DaS) and during three follow-up home-visits at 3, 6 and 12 months after discharge, using pre-stroke, clinical and healthcare information of the patient to analyse their HR QoL evolution over the first year post-stroke. Similar to the use of the total score of BI, the EQ-5D-3L index value also masks, however, the details of the patient health profile. For this, our second goal in this study is to apply neural network-based MTL to predict all the future answers of EQ-5D-3L survey of the patient while preserving the multi-level characteristic of each EQ-5D-3L dimension.

## 3 Datasets of patients with stroke

In this study, three datasets were used. The first one is the BI dataset for training models to predict a set of BI items of patients with stroke at 3 months after the onset of stroke symptoms (3MaS). The other two datasets were extracted from the clinical IST-3 trial (51, 52). Using these two datasets, the proposed MTL models were trained to predict the items of EQ-5D-3L questionnaire of patients at 6MaS and at 18MaS.

### 3.1 Barthel index data: Dataset 1

The first dataset relating to BI assessments of patients with stroke during their rehabilitation was collected at the Institut Guttmann (Barcelona, Spain) from 2002 to 2021.

#### 3.1.1 Barthel index

The BI (18) is a measure that was developed to evaluate the basic issues in ADLs of patients with musculoskeletal and neuromuscular pathologies, such as stroke. It scores the level of functional independence of patients along 10 items, namely Feeding, Bathing, Grooming, Dressing, Bowel control, Bladder control, Toilet Use, Transfers bed to chair and back, Mobility on Level Surfaces and Stairs.

BI items are scored using a two-, three- or four-point scale coded in steps of 5 points (i.e., the points on a two-point scale are 0 and 5; three-point scale 0, 5, 10; and four-point scale 0, 5, 10, 15). The higher the score the more independent the patient is on completing the ADL task. The minimum item score is 0, corresponding to a high dependence on help from the patient when performing the ADL task. The total BI score is calculated by summing up all the item scores. This total BI score can range from 0 to 100. The details of BI items and their scoring are shown in Supplementary Table S1.

As the follow-up BI items contain HR items of a patient with stroke, this set of items is considered as the target set that the proposed models will be trained to predict.

#### 3.1.2 Data source and preparation

The total number of patients in the BI dataset collected in the period under study (2002–2021) from the Institut Guttmann (Barcelona, Spain) is 1172. For this study, only patients who were admitted to the unit with a diagnosis of ischemic stroke were included. In line with previous related work on predicting stroke rehabilitation outcomes (such as the study of Scrutinio et al. (53)), we used a 3-month cutoff from stroke onset to discharge of rehabilitation as an inclusion criterion for this study. Patient's data includes gender, age at stroke, type of ischemic stroke, civil status, the number of days since stroke onset to admission, a National Institutes of Health Stroke Scale (NIHSS) (54) and a BI assessment on admission, and a BI assessment at discharge.

We filtered the dataset to only those patient records that included both:

1. a BI admission assessment completed within 45DaS, and
2. a BI discharge assessment completed between 70 and 120DaS.

**Figure 1:**
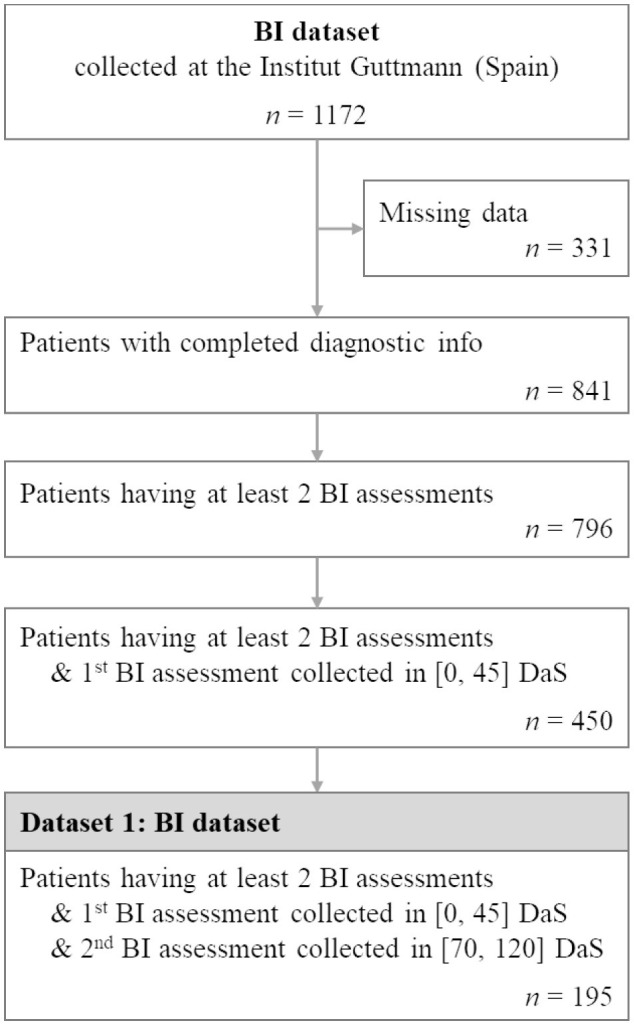
Flowchart illustrating patient selection for extracting BI dataset from the BI cohort collected at the Institut Guttmann. *n* represents the number of patients.

These number of days cut-offs were selected so that the timespan from admission to discharge was relatively consistent across patients while at the same time maximizing the final number of patients of the final filtered dataset. This filtering resulted in a dataset of 195 patient records which we used for our experiments. Figure 1 presents the flowchart of the extraction of the BI dataset for this study. Annexe 1 and Supplementary Table S2 detail the summary statistics of the studied Guttmann dataset.

**Figure 2:**
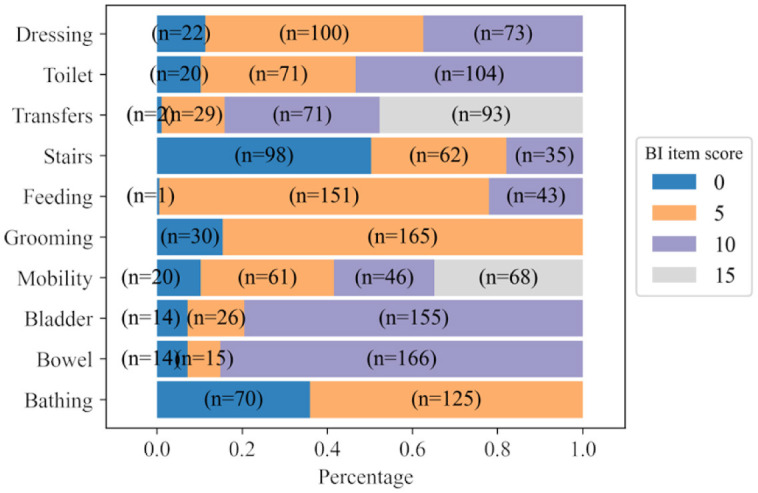
Distribution of scores for each BI item calculated from BI discharge assessments. *n* represents the number of patients.

Using the BI dataset, all demographics, diagnostics and BI data upon admission were used for predicting the BI item scores of the patients at discharge. Figure 2 shows the distribution of scores by items across the BI discharge assessment. The figure shows that the distribution of scores for BI items at discharge is imbalanced. There is 1 patient with a score of 0 for the BI item Feeding and only 2 patients with a score of 0 for the BI item Transfer. However, in keeping our goal of developing an MTL model for predicting the full BI set, we decided to keep all of these items as the target outputs.

### 3.2 IST-3 clinical trial data: dataset 2 and 3

The third international stroke trial (IST-3) dataset is a pragmatic international, multicentre, randomized, open-treatment trial which contains 3035 patients with stroke enrolled by 156 hospitals in 12 countries (51, 52). Eligible hospitals participating in IST-3 had to have an organized acute stroke service that met the local standard and guidelines of an effective stroke unit care.

Briefly, to meet the participation requirement a stroke unit should have a written protocol for the acute assessment of patients with suspected acute stroke to include interventions to reduce the time from onset to treatment, immediate access to computerized tomography (CT) or magnetic resonance imaging (MRI) brain scanning (24h a day), and a treatment area where thrombolysis may be administered and the patient monitored according to the trial protocol. The inclusion criteria of eligible patients in this trial were (i) patients had symptoms and signs of clinically definite acute stroke, (ii) the time of stroke onset was known, (iii) the treatment could be started within 6 hours of onset, and iv) the CT or MRI had reliably excluded both intracranial hemorrhage and structural brain lesions, which could mimic stroke (e.g., cerebral tumor).

In this trial data was recorded for each patient at admission to the trial (< 6 hours from onset of symptoms), at 24 h after stroke onset, at 7DaS, and at 6 and 18MaS. At admission the patient's demographic (e.g., age, gender, etc.) and diagnostic information (e.g., NIHSS, stroke type, comorbidities, blood tests, etc.) was recorded. At 24 hours after stroke, the acute treatment information for the patient was recorded. At 7DaS the patient's details regarding previous stroke, previous medication, medication administered between 24h to 7 days, and Glasgow coma scale (GCS) were recorded. At 6 and 18MaS the Oxford handicap score (OHS) as well as the EQ-5D-3L questionnaires were collected. This dataset is used to assess the ability of the MTL model to predict the EQ-5D-3L questionnaire responses for a patient at 6MaS and 18MaS.

#### 3.2.1 EQ-5D-3L

Developed by the EuroQoL group, EQ-5D (13, 14) is a commonly used instrument for measuring and comparing the health status of patients during rehabilitation (46). Originally, the EQ-5D was a five-dimensional three-level generic instrument. Nowadays, there are three different versions of EQ-5D measurements (46), the EQ-5D-3L (so-called EQ-5D or the 3-level version of EQ-5D) questionnaire is the first version, followed by EQ-5D-5L (the 5-level version of EQ-5D) and EQ-5D-Y (the child-friendly EQ-5D version). In our study, we use the EQ-5D-3L since the data of this questionnaire is available in the IST-3 data.

As shown in Supplementary Table S3, this instrument consists of 2 groups of information: the EQ-5D descriptive system and the EQ visual analog scale (EQ-VAS). The EQ-5D descriptive system has five dimensions (or items): Mobility, Self-care, Usual Activities, Pain/Discomfort, and Anxiety/Depression. Each dimension has 3 response categories namely “having no problem”, “having some problems” and “having extreme problems”. The EQ-VAS is a scoring scale that describes the overall health state of the patient going from 0 (as worst state) to 100 (as best state).

**Figure 3:**
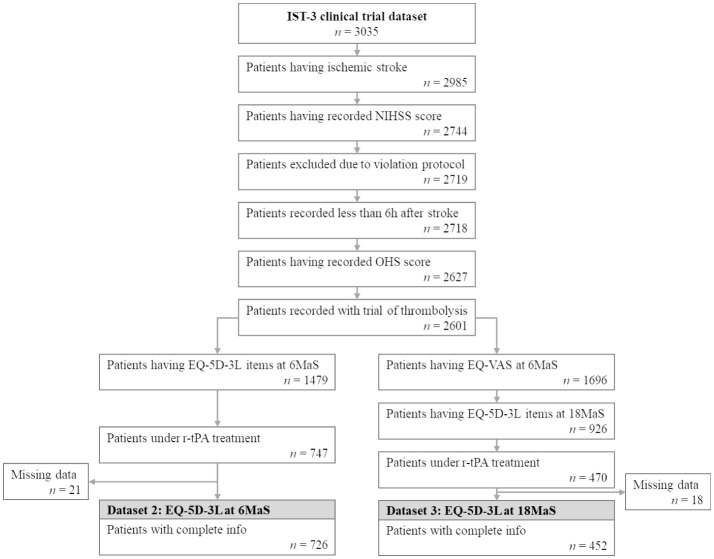
Flowchart illustrating patient selection for extracting EQ-5D-3L at 6MaS and at 18MaS datasets from the IST-3 clinical trial. *n* represents the number of patients.

Based on available follow-up information, two datasets are extracted for training models to predict the EQ-5D-3L of patients diagnosed with ischemic stroke at 6 and 18MaS. The common condition for extracting these two datasets includes only patients with ischemic stroke who are under recombinant tissue plasminogen activator (r-tPA) treatments. Data for patients that were added to the IST-3 trial more than 6h after stroke onset or whose record contains predicted NIHSS score, imputed OHS or was marked as violating protocol were excluded. Figure 3 presents the flowchart of the extraction of the two EQ-5D-3L datasets for this study.

#### 3.2.2 Dataset 2: EQ-5D-3L at 6MaS

For predicting the EQ-5D-3L set at 6MaS, all the information of patients from admission to the trial till 6MaS is used. Furthermore, we considered only the patients that are still alive at 6MaS, and for which all the EQ-5D-3L items collected at 6MaS are non-null. The final number of patients for the study of EQ-5D-3L at 6MaS is 726. The demographic, diagnostic information and follow-up information till 6MaS for this dataset are detailed in Annex 2 and Supplementary Table S4.

**Figure 4:**
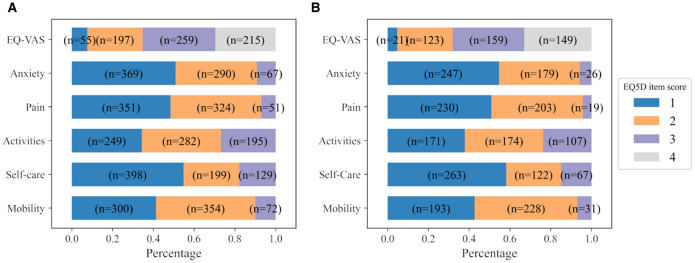
Distribution of scores of all EQ-5D-3L items at **(A)** 6MaS and **(B)** 18MaS. *n* represents the number of patients.

All the EQ-5D-3L items recorded at 6MaS are used as prediction targets. The distribution of scores of all EQ-5D-3L items collected at 6MaS is shown in Figure 4A. Here, the names of EQ-5D-3L items were shortened to ease the visualization. The EQ-VAS overall health score was binned into four equal categories and classified as 1 for a score in the interval of [1, 25], 2 for [26, 50], 3 for [51, 75], and 4 for [76, 100]. Together with the other five descriptive items, EQ-VAS binned scores are the set of output targets for training the QoL 6MaS model. More details about the variables used for training the QoL 6MaS model are shown in the Supplementary Table S5.

#### 3.2.3 Dataset 3: EQ-5D-3L at 18MaS

Similarly, the third dataset contains all the information of patients from admission to the trial till 18MaS. Furthermore, we considered only the patients that are still alive at 18MaS and for which all the EQ-5D-3L items collected at 6MaS and at 18MaS are non-null. The final number of patients for the study of HR profile at 18MaS is 452. Demographic, diagnostic information and follow-up information of this dataset are detailed in Annex 3 and Supplementary Table S6.

All the EQ-5D-3L items recorded at 18MaS are used as prediction targets for the personalized HR profile of the patients at 18MaS. The distribution of scores of all EQ-5D-3L items collected at 18MaS is shown in Figure 4B. Similar to the IST-3 6MaS dataset, the used EQ-VAS overall health scores were binned in four equal categories and classified as 1 for the score in the interval of [1,25], 2 for [26, 50], 3 for [51, 75] and 4 for [76, 100]. Then, the five descriptive items and the EQ-VAS binned scores are used as the output targets for training the QoL 18MaS model. More details about the variables used for training the QoL 18MaS model are shown in the Supplementary Table S7.

## 4 Data re-sampling methodology

Given the relatively small size of our datasets, we use a re-sampling (cross-validation) methodology for our experiments. Using cross-validation provides us with a method for assessing the variance of model performance across different splits of a dataset, thereby estimating the confidence in the mean performance as being a true estimate of model performance on the task.

The most commonly used form of cross-validation in ML is known as *k*-fold cross-validation where a dataset is split into *k* equal parts (or folds) and then each fold is used as a validation set for the model trained on the other *k*−1 folds. Using *k*-fold cross-validation, each data point occurs in exactly one validation set and *k*−1 training sets. Monte Carlo cross-validation is another well-known method where a test set and/or validation set is created by randomly sampling data points from the dataset without replacement. Once the test set and validation set of the desired size have been sampled the remaining data points are treated as the corresponding training set. The advantage of Monte Carlo cross-validation is that we can sample as many validation sets as we wish while maintaining a reasonable size for each validation set. Using Monte Carlo cross-validation some data points may occur in multiple validation sets and some may occur in none. However, this re-sampling method is still considered a cross-validation method as no data point occurs multiple times within a single validation or training set (which is not the case in a bootstrapping methodology).

**Figure 5:**
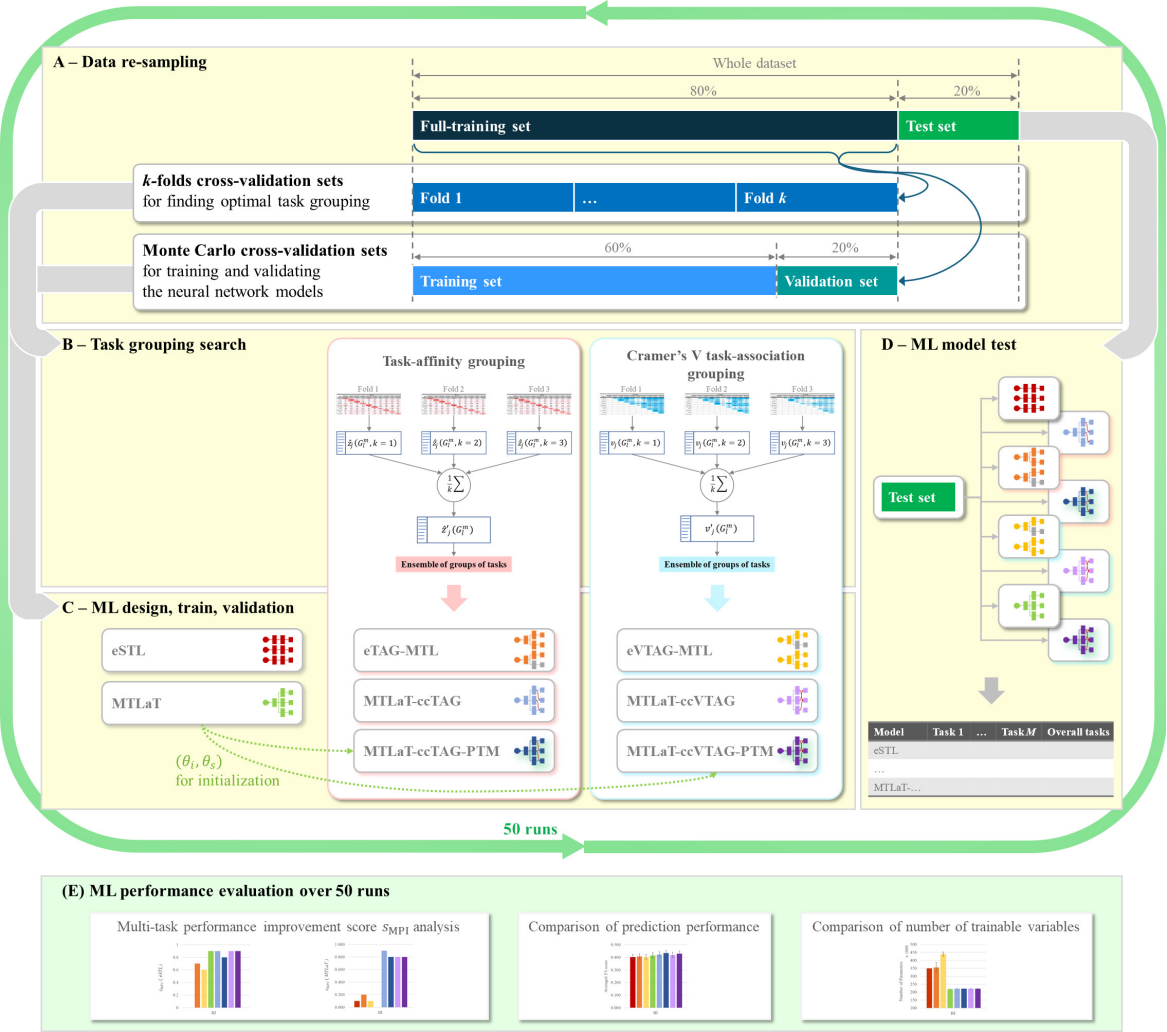
Flowchart of ML development for predicting HR profile. **(A)** Data re-sampling strategy used at each random split. **(B)** Task grouping search methodologies for finding optimal groups of tasks for MTL design in **(C)**, using data from the *k*-fold cross-validation sets of **(A)**. **(C)** ML model design for STLs and MTLs using the task grouping condition from **(B)**. STL and MTL models are then trained and validated using data from Monte-Carlo cross-validation sets in **(A)**. **(D)** Testing of trained STL and MTL models from **(C)** using the hold-out test set from **(A)**. **(E)** Evaluation of prediction performance of the studied ML models over 50 runs of ML development, each run is a cycle of **(A–D)**.

For each of our datasets (BI, EQ-5D-3L at 6MaS and 18MaS), we use Monte Carlo cross-validation to create 50 full-training and hold-out test sets containing 80% and 20% of the dataset.^1^ Each random split is generated using a random seed varied from 0 to 49. As shown in Figure 5A, for each of the 50 Monte Carlo splits we use the full-training data to do two things, first to determine the grouping of tasks that will be used in the MTL models (see Sections 5.3.2 and 5.4.2), and second to train the neural network models. To determine the task grouping, we use a *k*-fold cross-validation process on the full-training data (Figure 5B). Due to the limited size of the full-training splits, we used *k* = 3 for the BI dataset and *k* = 5 for EQ-5D-3L at 6MaS and 18MaS datasets. Once the task groupings have been determined, a corresponding MTL network model is trained and validated. This requires a second splitting of the full-training data (see Figures 5A, C). In this case, we again use Monte Carlo cross-validation to split the full-training data into training and validation sets of 75% and 25% of the full-training data (or equivalently 60% and 20% of the original dataset). Finally, the trained model is tested on the hold-out test set (Figure 5D). The performance of all the studied models is averaged over 50 runs for further evaluations (Figure 5E).

## 5 Methods: single- and multi-task learning

In this section, we introduce the different methods and modeling approaches that we use in our experiments. This includes STL, where a separate independent prediction model is trained for each task, the different methods we experiment with to identify related tasks (the task-affinity grouping and Cramer's V task-association grouping), and the strategies we propose and assess for constructing an MTL system.

### 5.1 Single-task learning

STL involves training a separate model to predict each output item. In this study, each STL model is a multilayer perceptron (MLP) feed-forward neural network (56) that consists of *h* hidden layers. Each hidden layer is composed of *r* nodes. Each of these nodes uses a Rectified Linear Unit (ReLU) activation function. The last hidden layer is then connected to a binary or multinomial output layer, depending on the studied output task.

For a binary task, the output layer contains a sigmoid activation unit and is trained with a binary cross-entropy loss. For a multinomial output layer, we use a softmax activation function, and this is trained with a categorical cross-entropy loss. All the STL models are trained and are updated using the sum of all losses of all units of the output layer (as described in Keras documentation (57)).

**Figure 6:**
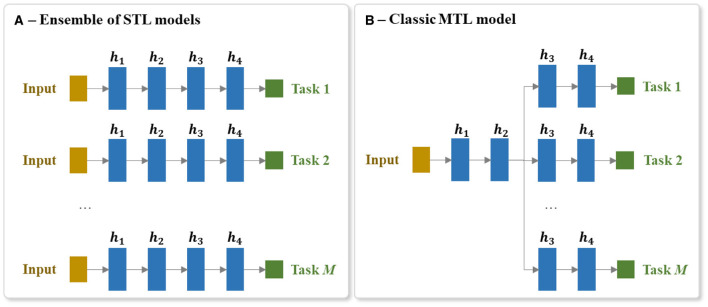
Architecture of the **(A)** ensemble of STL models and the **(B)** MTL model for predicting a set of *M* tasks. Each hidden layer is annotated as *h*_*i*_, where *i* is the *i*^th^ hidden layer of the network and *M* is the number of output tasks.

In order to use STL to create a system that can predict a set of tasks, we create an ensemble of STL models. Each STL model of the ensemble is independently trained to predict one output task. Figure 6A presents a brief visualization of the architecture of an ensemble of *M* STL models for predicting *M* output tasks.

### 5.2 Classic multi-task learning

MTL involves predicting multiple output items at the same time (e.g., predicting the scores of all the BI items). MTL is different to the more common multiclass prediction. Multiclass prediction involves classifying an example into one of three or more classes. When using a neural network, multiclass models are typically implemented using a softmax output layer. It is not possible, however, to scale a single softmax layer to MTL because a softmax layer only returns one output per instance. This is why, as mentioned above, the standard STL approach to handling a multitask prediction problem is to train a separate model per task. The drawback of this multimodel STL approach is that the separate models cannot share the useful information gleaned from related tasks being predicted in parallel. Consequently, to implement an MTL neural model that can share information between tasks, it is necessary to create a model that has multiple output layers (one output layer per task) and several shared layers of neurons which allows useful information to flow between the tasks. It also requires the integration of error gradients across multiple tasks during backpropagation through these shared layers. Figure 6B shows an overview of an MTL architecture for predicting *M* output tasks.

A classic MTL neural network model has at least a single shared layer across the different tasks followed by a set of task-specific output layers. In our experiments, a *h*-hidden-layer MTL of *M* tasks consists of a shared trunk of $\frac{h}{2}$ hidden layers connected to a set of *M* branches of $\frac{h}{2}$ hidden layers. Each hidden layer is composed of *r* nodes and each of these nodes uses a ReLU activation function. The last hidden layer of each of these *M* branches is then connected to its corresponding output layer.

Similar to the STL, binary output layers contain a sigmoid activation unit and are trained with a binary cross-entropy loss. Multinomial output layers use a softmax activation function and are trained with a categorical cross-entropy loss. MTL models are trained and are updated using the sum of all losses of all outputs (as described in Keras documentation (57)).

### 5.3 Multi-task learning with task-affinity grouping

The most straightforward approach to applying MTL to develop a prediction model for a set of targets is to train a single MTL model to predict all the items. The drawback with this approach, however, is that training a model to predict items in parallel that are not similar or share similar underlying structures can result in a reduction in model performance on one or more items (28, 38). Here, the goal of MTL with task-affinity grouping (TAG) (38) is to identify sets of tasks that can benefit from being learned in parallel. The selection of task grouping is done using the TAG measurement obtained by analyzing the training dynamics of an initial MTL model that learns all of the studied tasks at the same time.

#### 5.3.1 Task-affinity grouping

TAG is a non-symmetric^2^ pair-wise affinity measure between tasks where a high affinity of task *i* with task *j* indicates that learning task *i* in parallel with task *j* is beneficial in terms of learning task *j*. The calculation of task affinity is based on the intuition that if learning task *i* is beneficial for learning task *j* then back-propagating the losses on task *i* through the shared parameters of an MTL network should result in a reduction of the network's error on task *j*. In other words, if learning task *i* in parallel with task *j* will help to improve model performance in predicting task *j*, we should expect that if we save the state of the network during the training of a model that is learning task *i* and *j* in parallel, and then update the weights of the network using a loss signal from just task *i*, this should result in the performance of the updated network on task *j* being better than the performance of the saved network on task *j*. The larger the improvement on task *j* caused by updating the network parameters using the loss on task *i* the larger the affinity from task *i* to task *j*.

Consequently, TAG is calculated based on the change in the loss on one task (e.g., task *j*) before and after applying the gradient descent update from the other task (e.g., task *i*) onto the shared parameters. Following (38), we use the notation as below:

- θ = {θ_*s*_}∪{θ_*i*, 1 ≤ *i* ≤ *M*_} are the parameters of the considered multi-task loss function, where θ_*s*_ represents the shared parameters and θ_*i*_ represents the specific parameters of the *i*^th^ task and *M* is the number of tasks,
- *T* is the total number of training steps (or epochs),
- η is the learning rate,
- *L*_*i*_(χ, θ_*s*_, θ_*i*_) is the non-negative loss of task *i* for a given batch of instances χ,
- *L*_*j*_(χ, θ_*s*_, θ_*j*_) is the non-negative loss of task *j* for a given batch of instances χ,
- $\Delta_{\theta_{s|i}^{t}}$ is the gradient/derivative of error with regard to the shared parameters θ_*s*_ based solely on the loss for task *i* (i.e., *L*_*i*_(χ, θ_*s*_, θ_*i*_)) at the epoch *t* (1 ≤ *t* ≤ *T*, *T* is the maximum number of epochs),
- $\theta_{s|i}^{t+1}$ is the updated shared parameters after one step of backpropagation of $\Delta_{\theta_{s|i}^{t}}$,
- $L_{j|i}^{t+1}\left(\chi ,\theta_{s|i}^{t+1},\theta_{j}\right)$ is the non-negative lookahead loss of task *j* for a given batch of instances χ, when the shared parameters of the network (θ_*s*_) have been updated using the loss from the task *i* and χ (i.e., $\Delta_{\theta_{s|i}^{t}}$)
- $z_{i\to j}^{t}$ is the affinity of task *i* at a given training time-step *t* on task *j*, and is defined as:

(1) $$\begin{array}{l} z_{i\to j}^{t}=1-\frac{L_{j|i}^{t+1}\left(\chi ,\theta_{s|i}^{t+1},\theta_{j}\right)}{L_{j}\left(\chi ,\theta_{s},\theta_{j}\right)} \end{array}$$

A large positive value for $z_{i\to j}^{t}$ indicates that updating the network shared parameters using the loss of task *i* improves the model performance on *j*. Conversely, a large negative value for $z_{i\to j}^{t}$ indicates that updating the shared parameters with the loss of task *i* has a negative impact on the prediction of task *j*. A value for $z_{i\to j}^{t}$ close to zero means that updating using the loss of task *i* does not affect the performance on task *j*.

*z*_*i*→*j*_ defines the task affinity from task *i* onto task *j* at a single point during the training of a network. We generalize this affinity measure to the full training cycle of a network by defining ẑ_*i*→*j*_ as the average affinity of task *i* onto task *j* across multiple epochs of training.

**Figure 7:**
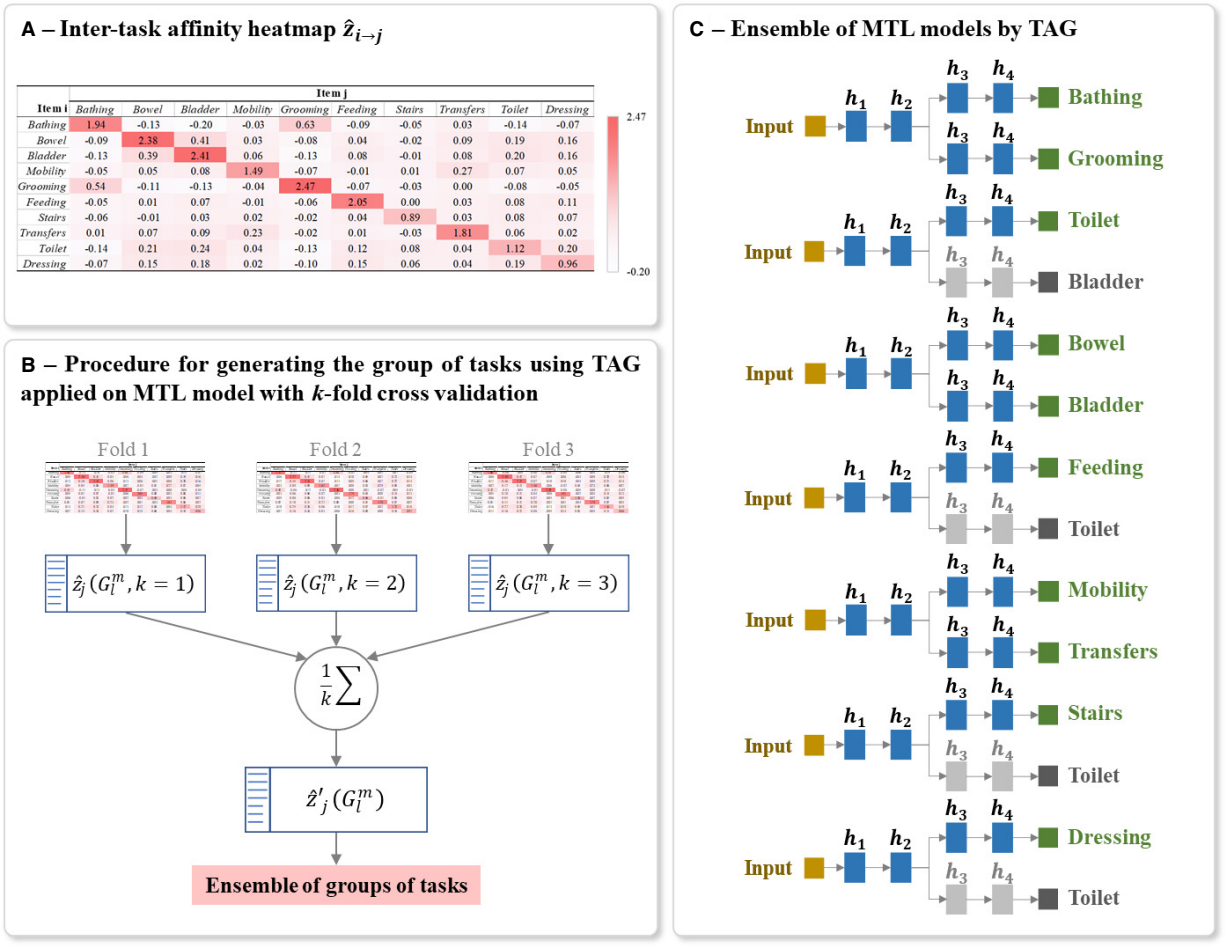
Example of **(A)** Heatmap of inter-task affinities ẑ_*i*→*j*_ of each task *i* on a given task *j* calculated over *T* training epoch, **(B)** Procedure for generating the optimal ensemble of task grouping from 3-fold cross-validation, and **(C)** Final ensemble of TAG-based MTL models. The inter-task affinities ẑ_*i*→*j*_ in **(A)** were calculated using the first fold of the full-training set of BI data. The architecture of **(C)** was generated from the ensemble of groups of tasks in **(B)**. The random seed used for this example is 42. The grayed branch output items are the secondary branch. *h*_*i*_ represents the *i*^th^ hidden layers.

The procedure for calculating the ẑ_*i*→*j*_ is detailed in the Algorithm 1. This algorithm is based on the approach proposed by (38). A ẑ_*i*→*j*_ is calculated for each possible pair of tasks. It should be noted that the learning rate is decreased during the training. This decreases the changes of the parameters $\theta_{s|i}^{t+1}$ when updating (step 8 of Algorithm 1), which induces a decreased difference between $L_{j|i}^{t+1}$ and $L_{j}^{t}$, thus $z_{i\to j}^{t}$ tend toward 0. To get rid of this effect, at each epoch we scale $z_{i\to j}^{t}$ by dividing it by the learning rate η used during that epoch. The values of ẑ_*i*→*j*_ for a studied multi-task problem can be presented as a heatmap, for example, see the heatmap shown in Figure 7A.

**Algorithm 1:**
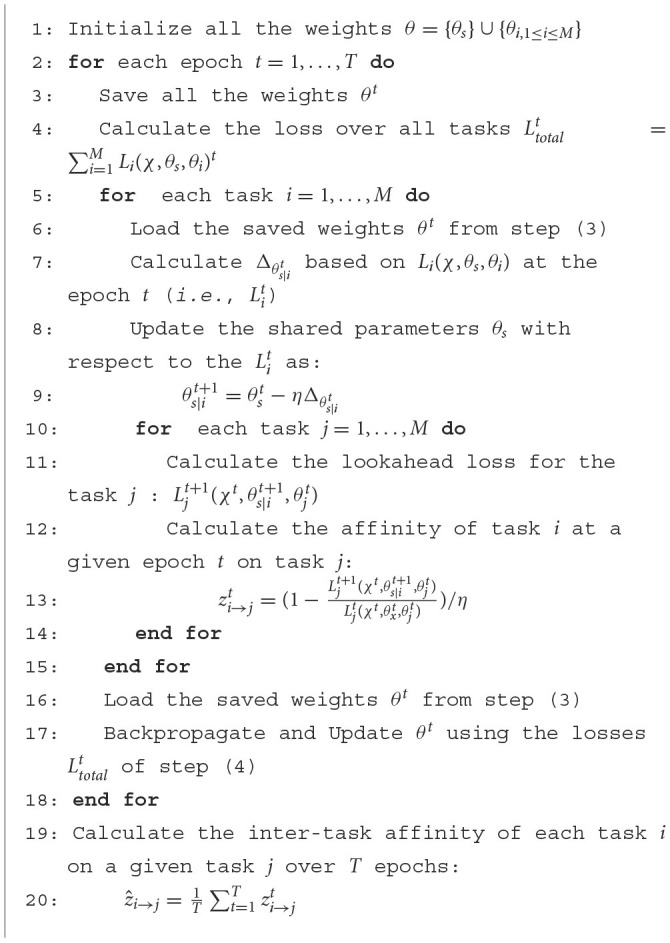
Inter-task affinity algorithm.

#### 5.3.2 TAG-based MTL network design

Once all the inter-task affinities ẑ_*i*→*j*_ have been calculated, the next step is to identify, for each task, the group of tasks it should be learned with to maximize model prediction performance on the task. By identifying these groups, we also define the set of MTL models that will be trained to predict the overall patient HR profile. In other words, the overall predicted patient HR profile is created by aggregating the predictions from a set of MTL models where each model is trained on a different group of items.

Since the number of tasks in one group is greater or equal to 2, the possible number of groups of tasks that we are selecting the MTL models from is calculated as $L=\overset{M}{\underset{m=2}{\sum }}$_*m*_*C*_*M*_, where _*m*_*C*_*M*_ denotes the combination or the number of possible selections of *m* tasks from the full set of *M* tasks, given that the order of selection is unimportant. *L* is the sum of all the combinations _*m*_*C*_*M*_ of varied number of tasks going from *m* = 2 to *m* = *M*. For the BI which had 10 ADL prediction target items, 1013 groups of tasks were studied, and for each of the IST-3 datasets of 6MaS and 18MaS (6 QoL items each), 57 groups were studied.

**The inter-task affinity onto a task** **j** **in a given group of tasks** (i.e., the affinity of the other tasks *i*≠*j* in the group in supporting the prediction of task *j*) is calculated as the mean of all the possible pairwise affinities on to this task. For the *l*^th^ group containing *m* tasks $G_{l}^{m}$ (where 2 ≤ *m* ≤ *M*, 2 ≤ *l* ≤ *L*), the inter-task affinity onto a task *j* is then :


(2) $$\begin{array}{l} {ẑ}_{j}\left(G_{l}^{m}\right)=\frac{1}{m-1}\overset{m}{\underset{i=1,i\neq j}{\sum }}{ẑ}_{i\to j}\left(G_{l}^{m}\right) \end{array}$$

To reduce the bias in generating the final architecture and to capture the maximum information from the full-training set, we applied *k*-fold cross-validation on this set for calculating inter-task affinity. As shown in Figure 7B, for each iteration of cross-validation, a heatmap of ẑ_*i*→*j*_ and a list of *L* group of tasks with its corresponding inter-task affinity ${ẑ}_{j}\left(G_{l}^{m}\right)$ are generated and averaged over the number of folds *k* as :


(3) $$\begin{array}{l} {ẑ}_{j}^{\prime }\left(G_{l}^{m}\right)=\frac{1}{k}\overset{k}{\underset{i=1}{\sum }}{ẑ}_{j}\left(G_{l}^{m},k\right) \end{array}$$

Using these averaged inter-task affinity ${ẑ}_{j}^{\prime }$, the optimal TAG-based MTL architecture is defined as follows. From the list of *L* groups, the group containing the task *j* which its corresponding inter-task affinity ${ẑ}_{j}^{\prime }\left(G_{l}^{m}\right)$ is the highest among the groups is denoted as $max\left({ẑ}_{j}^{\prime }\right)$. We then select all the groups $max\left({ẑ}_{j}^{\prime }\right)$ for all the *M* tasks and we include these groups in the final ensemble of models. This group selection process ensures that every task appears in at least one of the selected groups. A task may appear in more than one of the selected groups, but only the predictions for a task made by the MTL model trained on the group with the maximum inter-task affinity onto that task, i.e., the group $max\left({ẑ}_{j}^{\prime }\right)$, is used in the final aggregated prediction. The presence of a task in other groups indicates that its presence during training aids the learning of other tasks.

The final model for predicting the studied multiple tasks is thus an ensemble of MTLs where the grouping of tasks in each MTL is conditioned by TAG methodology. Each MTL model is constructed using the same parameters for structuring and training the classic MTL (Sections 5.2). Figure 7C shows an example of a final ensemble of TAG-based MTL models where its architecture was generated from the same split of the BI dataset. The branches containing grayed output items are only used to support the training of the MTL model. The predictions of these grayed-out branches are not used during inference.

### 5.4 Multi-task learning with Cramer's V task-association grouping

Similar to the MTL with TAG approach, the goal of MTL with Cramer's V task-association grouping (VTAG) is to identify sets of tasks that are related to each other and can benefit from being learned in parallel. Previous research has found that the performance of MTL is improved when there exists a dependency between the outputs (32, 33). Consequently, in this design of MTL systems, we carry out an analysis to identify groups of items that evince an association/dependency and we adopted the procedure of TAG-based MTL for generating the optimal MTL model with VTAG.

#### 5.4.1 Cramer's V: Dependence coefficient

In the literature, multiple methods can be used to assess the dependency between features such as Pearson's, Spearman's, or Cramer's V correlations (58, 59). In this design, we use Cramer's V to measure the dependency between the outputs of the tasks for designing an MTL model since this method is appropriate for nominal and especially for ordinal data (60), which is the data type of the BI and EQ-5D-3L features.

**Figure 8:**
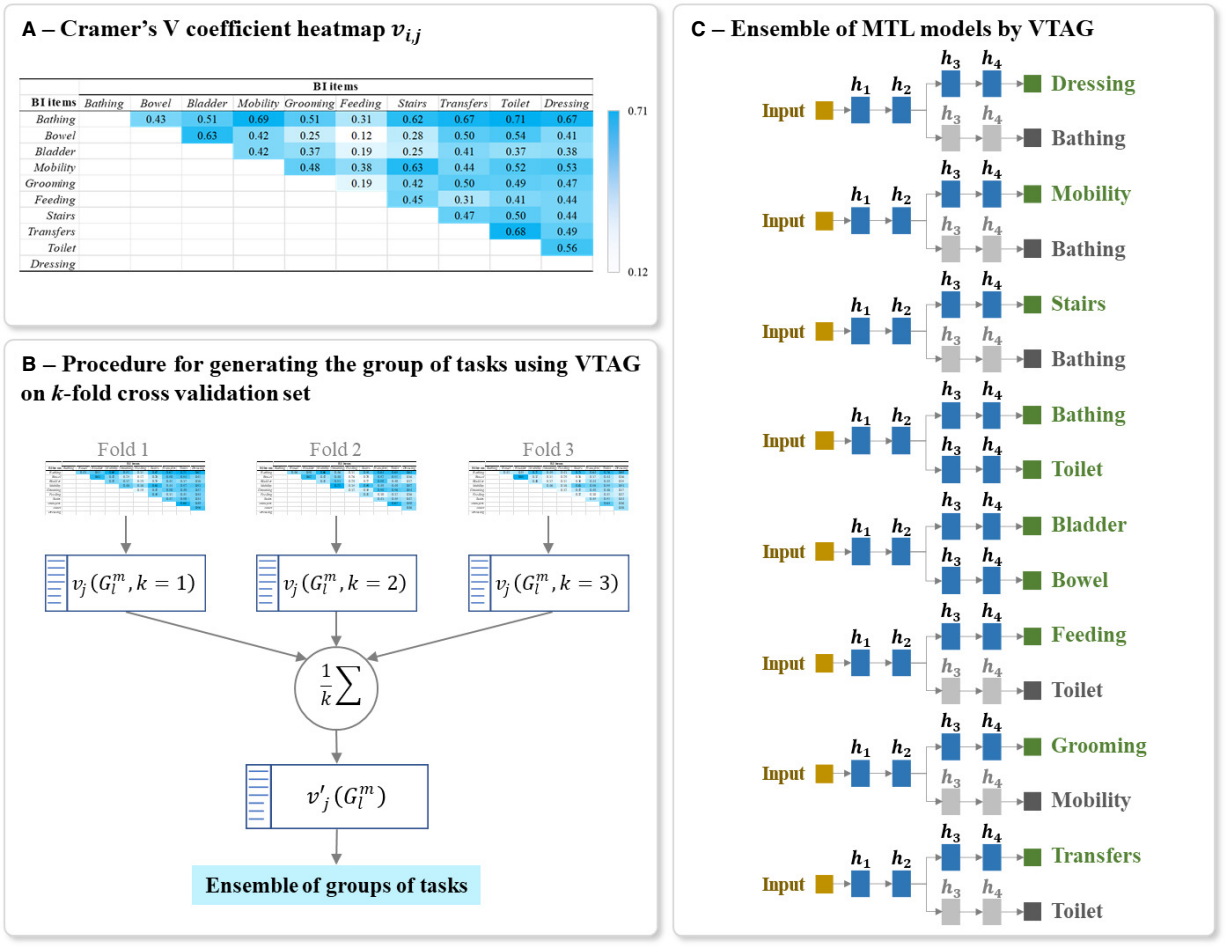
Example of **(A)** Heatmap of Cramer's V association *v*_*i, j*_ of all output item pairwise, **(B)** Procedure for generating the optimal ensemble of task grouping from 3-fold cross-validation, and **(C)** Final ensemble of VTAG-based MTL models. The Cramer's V coefficient heatmap *v*_*i, j*_ in **(A)** was calculated using the first fold of the full-training set of BI data. The architecture of **(C)** was generated from the ensemble of groups of tasks in **(B)**. The random seed used for this example is 42. The grayed branch output items are the secondary branch. *h*_*i*_ represents the *i*^th^ hidden layers.

By definition, Cramer's V (35, 36) is a statistical calculation developed to measure the strength of association between two nominal variables. Using this method, we first calculate a pairwise Pearson's chi-square statistical test between each pair of items to assess whether the strength of the relationship between pairs of output items is statistically significant. The Cramer's V coefficient is then calculated to measure the strength of association between each pair of items. We denote *v*_*i, j*_ as the Cramer's V coefficient value calculated for the pair of items (*i, j*), with 0 ≤ *i, j* ≤ *M* and *i*≠*j*. The value of *v*_*i, j*_ is positive and varying from 0, as no association, to 1, as very high association (36, 58). Also, *v*_*i, j*_ = *v*_*j, i*_ since Cramer's V is a symmetric pairwise association measure. The values of *v*_*i, j*_ for the studied HR profile items can be presented as a heatmap, see for example the heatmap shown in Figure 8A.

#### 5.4.2 Cramer's VTAG-based MTL network design

The design of a VTAG-based MTL network is inspired by the procedure of TAG-based MTL network design (as described in 5.3.2). Using the calculated *v*_*i, j*_, the inter-task association on to a task *j* in a given group of task $G_{l}^{m}$ is as follow:


(4) $$\begin{array}{l} v_{j}\left(G_{l}^{m}\right)=\frac{1}{m-1}\overset{m}{\underset{i=1,i\neq j}{\sum }}v_{i,j}\left(G_{l}^{m}\right) \end{array}$$

From the full training set, *k* Cramer's V heatmaps are generated from *k* training subsets of the *k*-fold cross-validation sets. As shown in Figure 8B, for each heatmap, a list of *L* groups of tasks is also extracted and the corresponding inter-task association of the *j*^th^ item in the group $G_{l}^{m}$ is averaged over *k* folds as:


(5) $$\begin{array}{l} v_{j}^{\prime }\left(G_{l}^{m}\right)=\frac{1}{k}\overset{k}{\underset{i=1}{\sum }}v_{j}\left(G_{l}^{m},k\right) \end{array}$$

Using these averaged inter-task associations $v_{j}^{\prime }$, the optimal VTAG-based MTL architecture is defined in the same way as for the TAG-based MTL, i.e., from the list of *L* possible groups of tasks, we select all the groups $max\left(v_{j}^{\prime }\right)$ for all the *M* tasks and we include those groups in the final ensemble of models.

The final model for predicting the studied multiple tasks is thus an ensemble of MTLs where the grouping of tasks in each MTL is conditioned by VTAG methodology. Each MTL model is constructed using the same parameters for structuring and training the classic MTL (Sections 5.2). Figure 8C shows an example of a final ensemble of VTAG-based MTL models where its architecture was generated from the same split of the BI dataset as used with TAG (Figure 7). The branches containing grayed output items are only used to support the training of the MTL model. The predictions of these grayed-out branches are not used during inference.

### 5.5 Multi-task learning with concatenation strategy

In both the TAG- and VTAG-based MTL methods described above, a separate MTL model is trained for each grouping of items, and no information is shared across these models. Another approach to creating an MTL model is to first create a single MTL architecture for all the tasks (as per Figure 6B), and then to augment this architecture with extra connections that concatenate the task branches that belong to the groups identified using either TAG or VTAG.

Concatenation is a technique of fusing information that has been used in well-known deep neural network architectures such as DenseNet (61), or GoogLeNet (62) and was frequently applied in designing multi-modal deep neural network (63, 64). Concatenation has also been used in MTL (e.g. for Alzheimer's disease progression detection (65) or for skin cancer diagnoses (66)) to fuse information derived from the inputs of different modalities. By adopting this concatenation strategy into our neural network design, the main structure and the parameters of the MTL model (such as, the output tasks, the number of hidden layers, the number of ReLUs per layer, the used activation for each output, etc.) are the same as described in Section 5.2. In addition to this architecture, the last hidden layer of the independent branches is concatenated together prior to connecting to the output layer.

**Figure 9:**
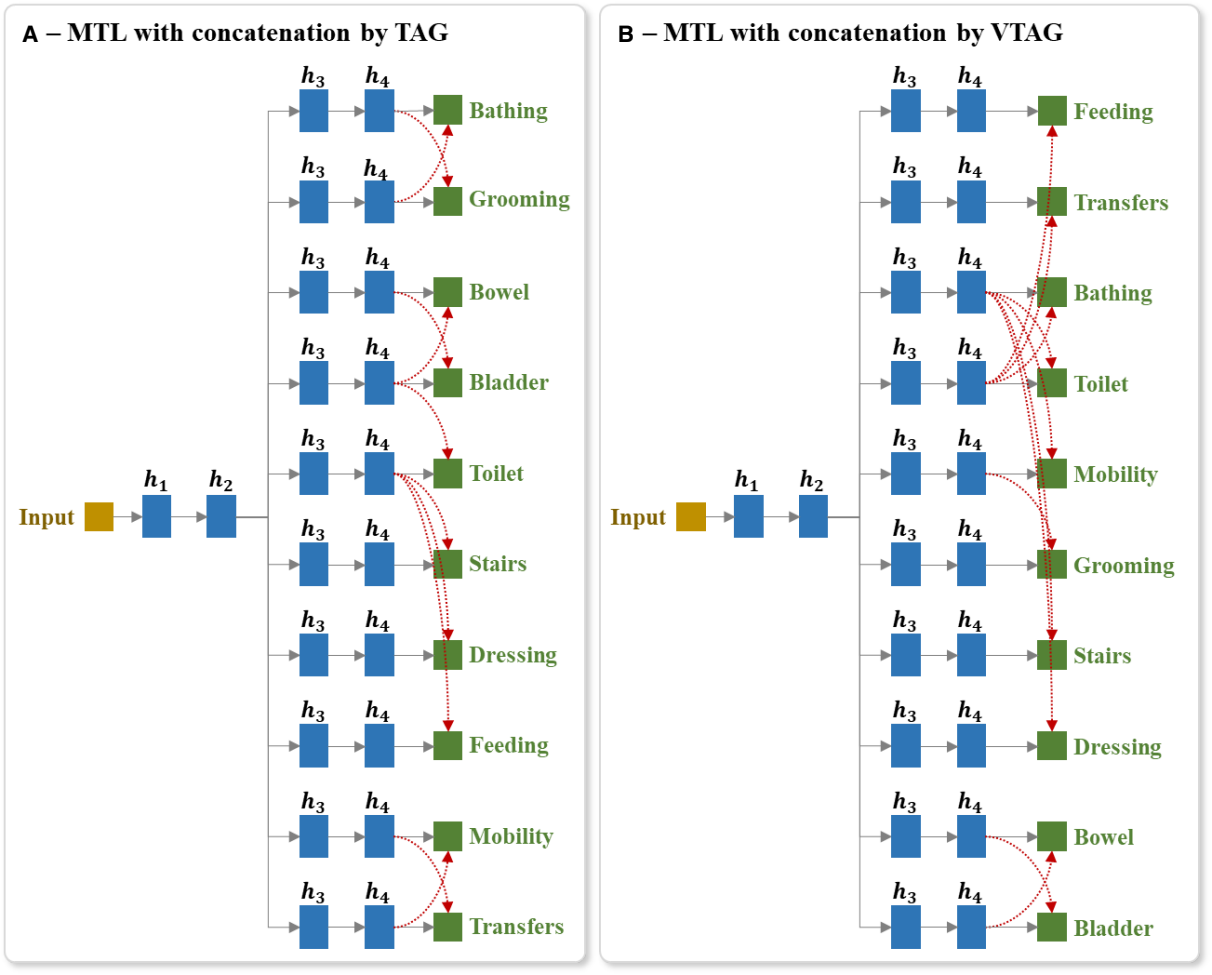
Example of the architecture of MTL of all tasks with concatenation between last hidden layers of output branches using **(A)** TAG and **(B)** VTAG grouping conditions. The red dashed arrows represent the concatenation links of the last hidden layers to the outputs. TAG and VTAG grouping are generated using the full-training set of the BI data, with random seed 42. *h*_*i*_ represents the *i*^th^ hidden layers.

For the MTL with TAG-based concatenation strategy, the concatenations are conditioned by the results of TAG methodology (as mentioned in Section 5.3.2). For each group of tasks $max\left({ẑ}_{j}^{\prime }\right)$, if the task *j* in the group is used for prediction, we link the last hidden layer of the branches corresponding to all the tasks of this group to the output *j*. Similarly for the MTL with VTAG-based concatenation strategy, the concatenations are conditioned by the results of VTAG grouping (as described in Section 5.4.2). Figures 9A, B respectively show an example of the architecture of the model MTL with TAG- and VTAG-based concatenation strategy, where ***h***_4_ represent the last hidden layer of the independent branch of each output. Those layers ***h***_4_ are concatenated and linked to the output layer for predicting the BI items (red links). This example architecture is designed using the BI dataset split generated from a random seed of 42.

Using this proposed architecture, we studied 2 training techniques. In the first technique, after the concatenation layers have been added, the whole network is initialized with random weights and is then trained. In the second technique, prior to the addition of the concatenation layers, we trained the MTL model for all tasks. We then added the concatenation layers and initialized these new layers with random weights but retained the trained weights in the other layers (i.e., we retain the trained weights from the pre-trained model (PTM)), and then trained the extended network by applying updates to all layers. The motivation for experimenting with this second training approach is that the TAG methodology for identifying groups of items to train together is based on the analysis of the dynamics of an MTL model trained on all tasks in parallel. Consequently, an interaction may exist between the groups identified using TAG and the weights of the model of the classic MTL of all tasks. Experimenting with both of these methods for initializing the network allows us to examine whether such an interaction exists.

### 5.6 Neural network development settings and evaluation metrics

In this work, each neural network was constructed with *h* = 4 hidden layers and each hidden layer contains *r* = 100 nodes. We used a stochastic gradient descent optimizer with a learning rate of η = 0.0005 and a momentum of 0.9. The learning rate is decreased by half after every 15 epochs. Early stopping is applied after 15 epochs without any improvement in loss value of validation and the waiting time is after at least 10 epochs have been run. Every network was trained with a maximum number of *T* = 200 epochs.

As discussed in Section 4, all experiments were run with 50 random seeds and for each random seed, one model of each model type (see Sections 5.1–5.5) is created. For each random seed, 20% of the dataset is randomly selected and kept out for testing, and 60% and 20% of the dataset are respectively used to train and validate the model. All data was normalized prior to modeling: continuous input features (e.g., age at stroke, NIHSS score, etc.) were normalized to [0,1] and categorical input features (e.g., gender, civil status, etc.) were one-hot-encoded.

The evaluation metric used in this work is the F1-score which is the harmonic average of precision and recall (29). For the multinomial output feature, the arithmetic average of the F1-score over the individual F1-score of each class was used as recommended by (67). To evaluate the performance of the studied neural network models, the arithmetic “average F1-score overall output tasks (or items)” was then calculated for each model.

Finally, to assess the performance of a neural network model against another we compare the F1-score of each output item predicted by that model *versus* the other one, e.g., the F1-score of Bathing of BI predicted by a classic MTL of all tasks model against the ensemble of STL models. We then calculate the ratio between the number of tasks that the MTL model outperformed STLs over the total number of tasks. This so-called *multi-task performance improvement (MPI) score* (*s*_MPI_) is varied from 0 to 1; where *s*_MPI_ = 0 represents no improvement in the prediction of MTL model comparing to STLs, and *s*_MPI_ = 1 indicates that the MTL has better performance than the STL on all tasks.

## 6 Results

We structure the reporting of our results by dataset. We begin by reporting our results on the prediction of the BI items, first by comparing single-task modeling with multi-task modeling and then extending the analysis to include multi-task modeling combined with concatenation approaches. We then report the results for the different modeling approaches when applied to the prediction of the EQ-5D-3L questionnaire of patients at 6 and 18MaS. As our experiments compare a large number of different modeling approaches here we define for each modeling strategy a label that we will use to refer to it throughout the presentation of the results.

- eSTL as the ensemble of STL models;
- eTAG-MTL as the ensemble of TAG-based MTL models;
- eVTAG-MTL as the ensemble of VTAG-based MTL models;
- MTLaT as the classic MTL of all tasks;
- MTLaT-ccTAG as MTL of all tasks with concatenation conditioned by TAG, trained from the initialization;
- MTLaT-ccTAG-PTM as MTL of all tasks with concatenation conditioned by TAG, trained from the PTM by loading the parameters from the already trained classic MTL of all tasks;
- MTLaT-ccVTAG as MTL of all tasks with concatenation conditioned by VTAG, trained from the initialization;
- MTLaT-ccVTAG-PTM as MTL of all tasks with concatenation conditioned by VTAG, trained from the PTM by loading the parameters from the already trained classic MTL of all tasks.

### 6.1 Prediction of BI items at 3MaS

This experiment aims to predict the patient's HR profile recorded by the BI assessment at 3MaS, based on their state at discharge. For this, demographic and clinical information, such as gender, age at stroke, type of ischemic stroke, civil status, NIHSS score, and BI items scores on admission of the patients are included in the dataset as model inputs. It should be noted that the BI total scores are not used as our focus is on predicting the individual BI items.

#### 6.1.1 Application of STL, classic MTL of all tasks, MTL with TAG and MTL with VTAG

The proposed models such as eSTL, eTAG-MTL, eVTAG-MTL and classic MTLaT were first trained to predict all the BI items. As shown in Table 1, the results of each method are averaged over the 50 results from the 50 data splits. The highest averaged F1-score overall BI items of 0.415 is found with MTLaT (standard deviation (STD) 0.021, confidence interval (CI) at 95% [0.409, 0.421]), which is statistically higher than the results obtained from eSTL, eTAG-MTL, and eVTAG-MTL. The second and the third best-performing models are respectively the eTAG-MTL and the eVTAG-MTL.

**Table 1:** Averaged F1-scores of the ensemble of STLs, ensemble of MTLs by TAG, ensemble of MTLs by VTAG and classic MTL of all tasks when trained on BI data.

	**BI item**											
**Model**	**Statistic**	**Bathing**	**Bowel**	**Bladder**	**Mobility**	**Grooming**	**Feeding**	**Stairs**	**Transfers**	**Toilet**	**Dressing**	**Overall (items)**
eSTL	Average	0.750	0.309	**0.298**	0.249	0.914	0.297	0.324	0.240	0.316†	0.325	0.402†
	STD	0.068	0.008	0.020	0.072	0.033	0.030	0.092	0.056	0.070	0.071	0.022
	CI	(0.73, 0.769)	(0.306, 0.311)	(0.292, 0.304)	(0.228, 0.269)	(0.905, 0.924)	(0.288, 0.305)	(0.298, 0.351)	(0.224, 0.256)	(0.296, 0.336)	(0.304, 0.345)	(0.396, 0.409)
eTAG-MTL	Average	**0.762**	0.308	0.298	0.253	0.915	0.294	0.325	0.252	0.327†	0.334	0.407†
	STD	0.056	0.008	0.021	0.065	0.034	0.025	0.091	0.053	0.076	0.061	0.021
	CI	(0.746, 0.778)	(0.306, 0.311)	(0.292, 0.304)	(0.234, 0.272)	(0.905, 0.924)	(0.287, 0.301)	(0.298, 0.351)	(0.236, 0.267)	(0.305, 0.349)	(0.317, 0.352)	(0.401, 0.413)
eVTAG-MTL	Average	0.755	0.308	0.298	0.247	0.915	0.297	0.313	0.243	0.322†	0.328	0.403†
	STD	0.054	0.008	0.021	0.064	0.034	0.026	0.089	0.052	0.068	0.065	0.020
	CI	(0.74, 0.771)	(0.306, 0.311)	(0.292, 0.304)	(0.229, 0.265)	(0.905, 0.925)	(0.289, 0.304)	(0.288, 0.339)	(0.228, 0.258)	(0.302, 0.341)	(0.309, 0.346)	(0.397, 0.408)
MTLaT	Average	0.758	**0.309**	0.296	**0.266**	**0.915**	**0.304**	**0.335**	**0.253**	0.368*	**0.350**	0.415*
	STD	0.053	0.008	0.012	0.069	0.034	0.036	0.084	0.055	0.068	0.060	0.021
	CI	(0.743, 0.773)	(0.306, 0.311)	(0.293, 0.299)	(0.246, 0.286)	(0.906, 0.925)	(0.294, 0.314)	(0.311, 0.359)	(0.237, 0.269)	(0.348, 0.387)	(0.333, 0.367)	(0.409, 0.421)

Results are averaged over 50 Monte Carlo data splits. Values in bold and grayed are the highest mean F1-score when comparing between models.

^*^Statistical significance of *t*-test between the F1-scores of MTL methodology and eSTL (*p* < 0.05).

^†^Statistical significance of *t*-test between the F1-scores of MTL methodology and MTLaT (*p* < 0.05).

STD, Standard deviation.

CI, Confidence interval at 95%.

**Figure 10:**
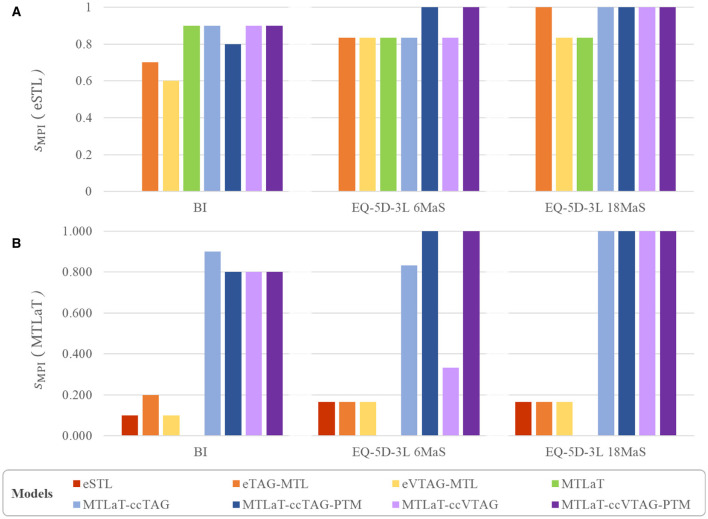
Multi-task performance improvement score (*s*_MPI_) of outperforming **(A)** eSTL and **(B)** MTLaT of prediction models when trained on BI, EQ-5D-3L at 6MaS and EQ-5D-3L at 18MaS datasets.

In this experiment, 8 out of 10 BI items were best predicted by MTLaT. Comparing the prediction of each item, the F1-scores of 9 (out of 10) items predicted by MTLaT were higher than those by eSTL, thus the MPI score of MTLaT is *s*_MPI_ = 0.9 (see Figure 10A). The models eTAG-MTL and eVTAG-MTL have *s*_MPI_ = 0.7 and 0.6, respectively. This result shows that MTLaT has the strongest MPI score of outperforming eSTL.

To summarize, our results indicate that MTL does improve prediction performance for BI items. More specifically, the MTLaT approach to MTL architecture design had the best overall performance, followed by eTAG-MTL, eVTAG-MTL, and eSTL.

#### 6.1.2 Application of MTL with concatenation strategies

The results from our first experiment on the BI indicated that the performances of the proposed eTAG-MTL and eVTAG-MTL are slightly higher than eSTL but this result is however not statistically significant (at the 95% CI). On the other hand, the classic MTLaT statistically outperforms these ensembles of MTL models. This indicates that the training of one model for predicting all the tasks at the same time is more powerful than training a small group of tasks, or even an independent task, separately. Also, given that the TAG approach for task grouping relies upon the training dynamics of the MTLaT model, there is potentially an interaction between the tasks of the groups selected by TAG and the weights of the MTLaT model. Inspired by this observation, in this section we assess the benefits of combining the MTLaT with a concatenation strategy applied to the last hidden layers. The selection of which task-specific branches to concatenate is based on the task groupings identified by the TAG or VTAG methodology (see Section 5.5). Each concatenation architecture is trained with and without retaining the weights from the pre-trained MTLaT model (PTM).

Table 2 lists the results obtained from this experiment. The data shows that the averaged F1-scores obtained by MTL with concatenation approaches are statistically significantly higher than eSTL. They also outperform MTLaT, especially the MTLaT-ccTAG-PTM and MTLaT-ccVTAG-PTM results are statistically higher than MTLaT. Among those trained models, the highest averaged F1-score is obtained with MTLaT-ccTAG-PTM of 0.434 (STD 0.022, CI at 95% [0.428, 0.44]). The performance of MTLaT-ccVTAG-PTM is the second highest with F1-score of 0.429 (STD 0.021, CI at 95% [0.423, 0.435]), followed by MTLaT-ccTAG and MTLaT-ccVTAG.

**Table 2:** Averaged F1-scores of the MTL of all tasks with concatenation conditioned by TAG and VTAG when trained on BI data.

	**BI item**											
**Model**	**Statistic**	**Bathing**	**Bowel**	**Bladder**	**Mobility**	**Grooming**	**Feeding**	**Stairs**	**Transfers**	**Toilet**	**Dressing**	**Overall (items)**
MTLaT	Average	0.758	**0.309**	0.296	0.266	**0.915**	0.304	0.335	0.253	0.368*	0.350	0.415*
	STD	0.053	0.008	0.012	0.069	0.034	0.036	0.084	0.055	0.068	0.060	0.021
	CI	(0.743, 0.773)	(0.306, 0.311)	(0.293, 0.299)	(0.246, 0.286)	(0.906, 0.925)	(0.294, 0.314)	(0.311, 0.359)	(0.237, 0.269)	(0.348, 0.387)	(0.333, 0.367)	(0.409, 0.421)
MTLaT-ccTAG	Average	**0.771**	**0.309**	0.296	0.270	0.915	0.313*	0.357	0.254	0.373*	0.362*	0.422*
	STD	0.045	0.008	0.012	0.069	0.033	0.048	0.083	0.052	0.068	0.066	0.022
	CI	(0.758, 0.784)	(0.306, 0.311)	(0.293, 0.3)	(0.25, 0.29)	(0.905, 0.925)	(0.299, 0.327)	(0.334, 0.381)	(0.239, 0.269)	(0.353, 0.392)	(0.343, 0.381)	(0.416, 0.428)
MTLaT-ccTAG-PTM	Average	0.767	0.309	**0.300**	0.282*	0.914	0.323*	0.395*,†	0.282*,†	0.389*	0.378*,†	0.434*,†
	STD	0.053	0.008	0.026	0.062	0.033	0.057	0.098	0.056	0.063	0.064	0.022
	CI	(0.752, 0.783)	(0.306, 0.311)	(0.292, 0.307)	(0.264, 0.3)	(0.905, 0.924)	(0.307, 0.34)	(0.367, 0.423)	(0.266, 0.299)	(0.371, 0.407)	(0.359, 0.396)	(0.428, 0.44)
MTLaT-ccVTAG	Average	0.765	**0.309**	0.296	0.264	**0.915**	0.305	0.347	0.252	0.375*	0.357*	0.419*
	STD	0.048	0.008	0.012	0.065	0.034	0.045	0.090	0.059	0.056	0.065	0.020
	CI	(0.751, 0.779)	(0.306, 0.311)	(0.293, 0.3)	(0.246, 0.283)	(0.906, 0.925)	(0.292, 0.319)	(0.321, 0.373)	(0.235, 0.269)	(0.359, 0.391)	(0.339, 0.376)	(0.413, 0.424)
MTLaT-ccVTAG-PTM	Average	0.767	0.308	**0.300**	0.277*	0.915	0.319*	0.381*,†	0.273*	0.392*	0.363*	0.429*,†
	STD	0.050	0.008	0.026	0.067	0.033	0.056	0.097	0.055	0.056	0.059	0.021
	CI	(0.753, 0.781)	(0.306, 0.311)	(0.292, 0.307)	(0.258, 0.297)	(0.905, 0.924)	(0.303, 0.335)	(0.353, 0.409)	(0.257, 0.288)	(0.376, 0.408)	(0.346, 0.38)	(0.423, 0.435)

The results of MTLaT is added for ease of comparison. Results are averaged over 50 Monte Carlo data splits. Values in bold and grayed are the highest mean F1-score when comparing between models.

^*^Statistical significance of *t*-test between the F1-scores of MTL methodology and eSTL (*p* < 0.05).

^†^Statistical significance of *t*-test between the F1-scores of MTL methodology and MTLaT (*p* < 0.05).

STD, Standard deviation.

CI, Confidence interval at 95%.

Combining the Tables 1, 2, obtained results show that 6 out of 10 BI items are best predicted by MTLaT-ccTAG-PTM. As shown in Figure 10A, the MPI score of outperforming eSTL of the models of MTL of all tasks are high: MTLaT, MTLaT-ccTAG, MTLaT-ccVTAG and MTLaT-ccVTAG-PTM have *s*_MPI_ = 0.9 and MTLaT-ccTAG-PTM has *s*_MPI_ = 0.8. However, the ensembles of MTL models of eTAG-MTL and eVTAG-MTL have low *s*_MPI_ scores of 0.6 and 0.7, respectively. For the MPI score of outperforming MTLaT (Figure 10B), the models of eSTL, eTAG-MTL and eVTAG-MTL have the lowest *s*_MPI_ ranging from 0.1 to 0.2, indicating that the prediction of the 80-90% of the output tasks are worse. On the other hand, MTLaT-ccTAG-PTM, MTLaT-ccVTAG and MTLaT-ccVTAG-PTM have *s*_MPI_ = 0.8 and MTLaT-ccTAG has *s*_MPI_ = 0.9. These results show that the MTLs with concatenation architectures outperform the reference eSTL and MTLaT models on more than 80% of the predicted output tasks.

To summarize, the averaged F1-scores of most of the proposed MTL with concatenation approaches are statistically higher than eSTL and MTLaT. Compared with the results of the other applications (i.e., STL, classic MTL of all tasks, MTL with TAG, and MTL with VTAG), the TAG-based MTL of all tasks with concatenation and PTM is the best model for predicting the set of BI items.

### 6.2 Prediction of EQ-5D-3L at 6MaS

For this second experiment, all the available demographic, clinical, diagnostic, and treatment information and the stroke outcome of the patients collected from the admission till 7DaS (or at hospital discharge or transfer if earlier) was used to train the model for predicting the details of the EQ-5D-3L responses at 6MaS. The prediction model can inform the clinician of the likely future QoL state of the patient (EQ-5D-3L at 6MaS) and help the clinician design the rehab treatment for the patient after 7DaS.

Table 3 shows the prediction performance of all the studied models when trained on the IST-3 data for predicting EQ-5D-3L profile at 6MaS. The obtained results show that MTLaT-ccVTAG-PTM is the best-performing model with the highest averaged F1-score of 0.388 (STD 0.029, CI at 95% [0.38, 0.397]). Similarly, MTLaT-ccTAG-PTM obtains the second highest averaged F1-score of 0.388 (STD 0.029, CI at 95% [0.379, 0.396]). All the MTL models have a statistically significant improvement in prediction performance compared with eSTL. Furthermore, MTLaT-ccTAG-PTM and MTLaT-ccVTAG-PTM outperform MTLaT by a statistically significant margin.

**Table 3:** Averaged F1-scores of the ensemble of STLs, TAG-based MTLs, VTAG-based MTLs, classic MTL of all tasks, MTL of all tasks with concatenation conditioned by TAG and VTAG with and without PTM when trained on IST-3 data of EQ-5D-3L at 6MaS.

	**EQ-5D-3L item**							
**Model**	**Statistic**	**Mobility**	**Self-care**	**Activities**	**Pain**	**Anxiety**	**EQ-VAS**	**Overall (items)**
eSTL	Average	0.341†	0.287†	0.359†	0.346†	0.316†	0.240	0.315†
	STD	0.055	0.044	0.061	0.038	0.039	0.034	0.027
	CI	(0.326, 0.357)	(0.275, 0.3)	(0.342, 0.377)	(0.336, 0.357)	(0.305, 0.327)	(0.23, 0.249)	(0.307, 0.323)
eTAG-MTL	Average	0.365*,†	0.306†	0.389*,†	0.357†	0.323†	0.238	0.33*†
	STD	0.053	0.054	0.062	0.037	0.041	0.039	0.029
	CI	(0.35, 0.38)	(0.29, 0.321)	(0.371, 0.407)	(0.346, 0.367)	(0.311, 0.335)	(0.227, 0.249)	(0.321, 0.338)
eVTAG-MTL	Average	0.366*,†	0.307*,†	0.391*,†	0.356†	0.32†	0.238	0.33*†
	STD	0.053	0.054	0.063	0.040	0.042	0.036	0.029
	CI	(0.351, 0.381)	(0.292, 0.323)	(0.373, 0.409)	(0.345, 0.368)	(0.308, 0.332)	(0.227, 0.248)	(0.321, 0.338)
MTLaT	Average	0.391*	0.335*	0.428*	0.383*	0.354*	0.236	0.355*
	STD	0.039	0.056	0.060	0.034	0.037	0.041	0.027
	CI	(0.38, 0.402)	(0.319, 0.351)	(0.411, 0.445)	(0.373, 0.393)	(0.344, 0.365)	(0.225, 0.248)	(0.347, 0.363)
MTLaT-ccTAG	Average	0.395*	0.373*,†	0.429*	0.385*	0.36*	0.235	0.363*
	STD	0.038	0.066	0.068	0.030	0.033	0.032	0.031
	CI	(0.384, 0.406)	(0.354, 0.392)	(0.41, 0.449)	(0.376, 0.393)	(0.35, 0.369)	(0.226, 0.244)	(0.354, 0.372)
MTLaT-ccTAG-PTM	Average	0.42*,†	0.427*,†	0.471*,†	0.396*,†	0.37*,†	**0.241**	0.388*,†
	STD	0.043	0.064	0.057	0.028	0.027	0.035	0.029
	CI	(0.408, 0.432)	(0.409, 0.446)	(0.455, 0.488)	(0.388, 0.404)	(0.362, 0.378)	(0.231, 0.251)	(0.379, 0.396)
MTLaT-ccVTAG	Average	0.386*	0.376*,†	0.432*	0.382*	0.353*	0.234	0.36*
	STD	0.041	0.064	0.068	0.030	0.035	0.034	0.032
	CI	(0.375, 0.398)	(0.358, 0.394)	(0.412, 0.452)	(0.373, 0.391)	(0.343, 0.363)	(0.224, 0.243)	(0.351, 0.37)
MTLaT-ccVTAG-PTM	Average	0.413*,†	0.44*,†	0.475*,†	0.395*	0.367*	0.240	0.388*,†
	STD	0.036	0.065	0.057	0.029	0.028	0.035	0.029
	CI	(0.403, 0.424)	(0.421, 0.458)	(0.459, 0.492)	(0.387, 0.404)	(0.359, 0.375)	(0.23, 0.25)	(0.38, 0.397)

Results are averaged over 50 Monte Carlo data splits. Values in bold and grayed are the highest mean F1-score when comparing between the all the studied models.

^*^Statistical significance of *t*-test between the F1-scores of MTL methodology and eSTL (*p* < 0.05).

^†^Statistical significance of *t*-test between the F1-scores of MTL methodology and MTLaT (*p* < 0.05).

STD, Standard deviation.

CI, Confidence interval at 95%.

Regarding the prediction performance of each item, Self-care and Activities are best predicted by MTLaT-ccVTAG-PTM and the remaining items (Mobility, Pain, Anxiety and EQ-VAS) are by MTLaT-ccTAG-PTM. The MPI score of outperforming eSTL of all the MTL models is in the range of [0.833, 1], showing that the prediction in 83% of the output tasks is improved when comparing to eSTL (see Figure 10A). Regarding the MPI score of outperforming MTLaT, the models of eSTL, eTAG-MTL and eVTAG-MTL have the lowest score *s*_MPI_ = 0.167 and MTLaT-ccVTAG has a score of *s*_MPI_ = 0.333 indicating that the prediction of the majority of the output tasks are worse (see Figure 10B). In contrast, MTLaT-ccTAG has a high score of *s*_MPI_ = 0.833. Especially, the two models of MTLaT-ccTAG-PTM and MTLaT-ccVTAG-PTM have the highest score of 1 when compared to eSTL or MTLaT.

In summary, we obtain similar results as with the BI data and STL is the least optimal architecture for predicting EQ-5D-3L at 6MaS, and the optimal model is MTLaT-ccVTAG-PTM. The model MTL of all tasks with concatenation and initialized by MTLaT weights gives the best results when compared to the classic STL and MTL of all tasks.

### 6.3 Prediction of EQ-5D-3L at 18MaS

Similar to the last experiment, all the available information and the stroke outcome of the patients collected from the admission till 6MaS was used to train the models to predict the patient EQ-5D-3L responses at 18MaS. The clinician can use this model to be informed about the likely future HR profile of the patient at 18MaS after collecting their EQ-5D-3L assessment at 6MaS, which can help the clinician design the rehab treatment and follow up on the progress of the treatment on the patient after 6MaS.

Table 4 shows the prediction performance of all the studied models when trained on IST-3 data of EQ-5D-3L profile at 18MaS. Here, MTLaT-ccTAG-PTM is the best-performing model with the highest averaged F1-score of 0.462 (STD 0.029, CI at 95% [0.454, 0.47]). MTLaT-ccVTAG-PTM is the model with the second highest averaged F1-score of 0.462 (STD 0.030, CI at 95% [0.453, 0.47]). The prediction of all the MTL models is statistically significantly higher than eSTL and compared to MTLaT, all the MTL models with concatenation strategy statistically significantly outperform MTLaT.

**Table 4:** Averaged F1-scores of the ensemble of STLs, TAG-based MTLs, VTAG-based MTLs, classic MTL of all tasks, MTL of all tasks with concatenation conditioned by TAG and VTAG with and without PTM application when trained on IST-3 data of EQ-5D-3L at 18MaS.

	**EQ-5D-3L item**							
**Model**	**Statistic**	**Mobility**	**Self-care**	**Activities**	**Pain**	**Anxiety**	**EQ-VAS**	**Overall (items)**
eSTL	Average	0.415†	0.305†	0.438†	0.375†	0.346†	0.267	0.358†
	STD	0.054	0.054	0.062	0.044	0.048	0.052	0.032
	CI	(0.4, 0.43)	(0.289, 0.32)	(0.42, 0.456)	(0.362, 0.388)	(0.332, 0.36)	(0.252, 0.282)	(0.348, 0.367)
eTAG-MTL	Average	0.448*,†	0.34*,†	0.472*	0.405*,†	0.37*,†	0.267	0.384*,†
	STD	0.043	0.071	0.057	0.043	0.052	0.052	0.034
	CI	(0.436, 0.461)	(0.319, 0.36)	(0.455, 0.488)	(0.393, 0.417)	(0.355, 0.385)	(0.252, 0.282)	(0.374, 0.393)
eVTAG-MTL	Average	0.445*,†	0.34*,†	0.474*	0.375†	0.345†	0.270	0.375*,†
	STD	0.042	0.072	0.060	0.041	0.053	0.052	0.035
	CI	(0.433, 0.457)	(0.319, 0.361)	(0.457, 0.491)	(0.363, 0.387)	(0.33, 0.36)	(0.255, 0.285)	(0.365, 0.385)
MTLaT	Average	0.476*	0.388*	0.475*	0.433*	0.414*	0.263	0.408*
	STD	0.042	0.071	0.057	0.042	0.044	0.050	0.031
	CI	(0.463, 0.488)	(0.368, 0.408)	(0.458, 0.491)	(0.421, 0.445)	(0.401, 0.427)	(0.248, 0.277)	(0.399, 0.417)
MTLaT-ccTAG	Average	0.478*	0.434*,†	0.499*	0.436*	0.416*	0.284†	0.424*,†
	STD	0.041	0.081	0.074	0.035	0.044	0.050	0.035
	CI	(0.466, 0.49)	(0.41, 0.457)	(0.478, 0.52)	(0.425, 0.446)	(0.403, 0.429)	(0.27, 0.299)	(0.414, 0.435)
MTLaT-ccTAG-PTM	Average	0.495*,†	0.517*,†	0.55*,†	0.451*,†	0.443*,†	0.315*,†	0.462*,†
	STD	0.037	0.080	0.063	0.031	0.035	0.051	0.029
	CI	(0.485, 0.506)	(0.494, 0.54)	(0.532, 0.568)	(0.442, 0.46)	(0.433, 0.453)	(0.3, 0.33)	(0.454, 0.47)
MTLaT-ccVTAG	Average	0.481*	0.436*,†	0.495*	0.434*	0.415*	0.282	0.424*,†
	STD	0.043	0.078	0.070	0.039	0.046	0.054	0.036
	CI	(0.469, 0.493)	(0.413, 0.458)	(0.475, 0.515)	(0.423, 0.445)	(0.401, 0.428)	(0.267, 0.298)	(0.413, 0.434)
MTLaT-ccVTAG-PTM	Average	0.496*,†	0.519*,†	0.552*,†	0.448*	0.44*,†	0.314*,†	0.462*,†
	STD	0.041	0.080	0.067	0.033	0.033	0.053	0.030
	CI	(0.485, 0.508)	(0.496, 0.542)	(0.533, 0.572)	(0.438, 0.457)	(0.43, 0.45)	(0.299, 0.329)	(0.453, 0.47)

Results are averaged over 50 Monte Carlo data splits. Values in bold and grayed are the highest mean F1-score when comparing between the all the studied models.

^*^Statistical significance of *t*-test between the F1-scores of MTL methodology and eSTL (*p* < 0.05).

^†^Statistical significance of *t*-test between the F1-scores of MTL methodology and MTLaT (*p* < 0.05).

STD, Standard deviation.

CI, Confidence interval at 95%.

For the prediction of each item, Mobility, Self-care, and Activities are best predicted by MTLaT-ccVTAG-PTM and the remaining items (Pain, Anxiety, and EQ-VAS) are by MTLaT-ccTAG-PTM. The MPI scores of outperforming eSTL of all the MTL models are in the range of [0.833, 1] as shown in Figure 10A. This suggests that the prediction of 83% of the output tasks is improved, especially eTAG-MTL model achieves an MPI score *s*_MPI_ = 1 indicating that this model outperforms eSTL on all tasks for predicting the EQ-5D-3L items at 18MaS. Regarding the MPI score of outperforming MTLaT, the models of eSTL, eTAG-MTL and eVTAG-MTL have the lowest *s*_MPI_ = 0.167. However, all the MTL models with concatenation strategies have the highest score of *s*_MPI_ = 1 (see Figure 10B).

In summary, we obtain similar results as the other experiments and eSTL is the least optimal architecture for predicting EQ-5D-3L at 18MaS and the optimal model is MTLaT-ccTAG-PTM. The models of MTL of all tasks with concatenation and initialized by MTLaT weights outperform the prediction of all the tasks when compared to the reference models eSTL and MTLaT.

### 6.4 Comparison between neural network models

**Figure 11:**
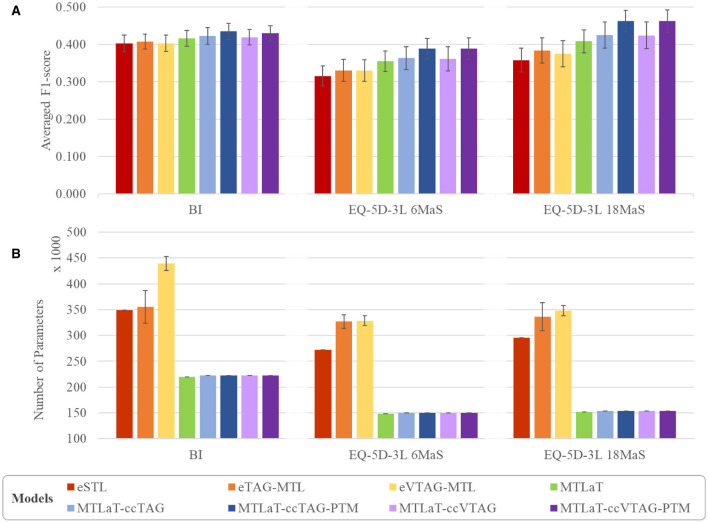
Comparison of **(A)** averaged prediction performance and **(B)** averaged number of trainable variable between the proposed models when trained on BI, EQ-5D-3L at 6MaS and EQ-5D-3L at 18MaS datasets. Error bars represent the standard deviation calculated over 50 random initializations.

To evaluate the performance of the studied neural architectures, we compare the performance of each architecture for each applied dataset. Figure 11A shows the multiple-bar plots of the mean performance of the eight architectures trained on BI, EQ-5D-3L at 6MaS and EQ-5D-3L at 18MaS datasets. For each dataset, each plotted value is calculated as the mean of the “average F1-scores over all the output items” of the given architecture. The corresponding STD is presented as the error bars in the same plot. Here, eSTL is shown to be the weakest architecture on any of the datasets. The next higher architectures are eVTAG-MTL and eTAG-MTL, where eTAG-MTL is slightly better than eVTAG-MTL.

For the green bar of MTLaT, this classic architecture of MTL outperforms the ensemble of STLs and MTLs. Similar results are obtained with all the architectures with concatenation strategy (i.e. MTLaT-ccTAG, MTLaT-ccTAG-PTM, MTLaT-ccVTAG and MTLaT-ccVTAG-PTM) which outperform the ensemble of STLs and MTLs. We have also found that the MTL with concatenation methodology tends to work best when we initialize the model with the pre-trained parameters from the corresponding MTLaT model. This can be seen in the blue and violet bars in the plot, especially the dark blue and dark violet are the highest in any dataset and represent respectively the mean performance of MTLaT-ccTAG-PTM and MTLaT-ccVTAG-PTM. Compared to eSTL, the use of a concatenation strategy with PTM improves performance by more than 6%, 23%, and 29% when training on BI, EQ-5D-3L at 6MaS and EQ-5D-3L at 18MaS, respectively; and when comparing with MTLaT the concatenation with PTM strategy improves performance by more than 3%, 9% and 13%.

Although our results indicate that the use of a concatenation strategy consistently improves performance on all datasets, the selection of which task grouping strategy (TAG or VTAG) will give the best results is dependent on the data. Especially for IST-3 data, the prediction performance between MTLaT-ccTAG and MTLaT-ccVTAG, and between MTLaT-ccTAG-PTM and MTLaT-ccVTAG-PTM are very comparable.

Figure 11B shows the average number of parameters of each of the studied neural network architectures, where each plotted value is the mean of the number of parameters of all studied networks. The corresponding standard deviation is also measured and presented as the error bars in the same plot (the details of the number of parameters for each model are shown in Supplementary Table S8). Here, MTLaT has the lowest number of parameters. eVTAG-MTL and eTAG-MTL have respectively the highest and second-highest number of parameters in these experiments.

It should be noted that the ensemble of groups of tasks generated by TAG and VTAG on the studied datasets are all ensembles of 2-tasks groups. However, the number of groups of tasks selected by VTAG is usually greater than by TAG resulting in the fact that eVTAG-MTL has a higher number of parameters than eTAG-MTL. Furthermore, some groups of tasks may contain tasks that are not used during inference but are only for supporting the training of other tasks in the group as part of the MTL process. This is why the number of parameters in models based on the eTAG-MTL and eVTAG-MTL architecture can grow. The models that have the third highest number of parameters is eSTL. Interestingly, the networks of MTL of all tasks (with and without concatenation) are shown to be the ones that have the fewest parameters while keeping the best performance on all the studied output tasks.

## 7 Discussion

The main clinical purpose of this work is to develop a prediction model that can predict a set of items of the HR profile, (i.e., a set of HR items either of QoL or of iADL) of a patient at the post-acute stage. The idea of predicting the score of each HR profile item is to show the clinician both the activities that the patient is likely to improve on and also the tasks that the patient is likely to struggle with. Identifying the activities that a patient will likely struggle with helps the clinician focus the post-stroke treatment program on the exercises and care that will help the patient improve on these ADL items.

For this, we initially used a dataset provided by the Guttmann Hospital and developed a methodology for creating models to predict the outcomes of BI items of the patient at 3MaS. All of the model architectures developed to predict BI were next trained on two different datasets of IST-3 for predicting the set of EQ-5D-3L items of patients at 6 and 18MaS. By gathering the results across the BI and IST-3 prediction tasks, we analyzed the benefit of using an MTL model for all tasks versus STL for predicting HR profiles. Then, we explored the advantages of different variants of MTL compared with the classic MTL and STL models.

When training the classic STL and MTL of all task models on the three studied datasets, the prediction performance of MTL of all tasks is consistently and statistically higher than STLs. Also, the MTL predictions have higher averaged F1-scores on the majority of tasks compared to the STL predictions. These results demonstrate that learning multiple tasks at the same time using MTL is beneficial for prediction, as previously published in the literature (7, 28).

Furthermore, recent MTL studies suggest that training an MTL model on selected groups of related tasks may help to improve the prediction (37, 38). Building on the initial framework for the prediction of structured outputs (24), we adopted inter-task affinity (for TAG) and Cramer's V (for VTAG) to calculate the inter-dependence between tasks and we use these metrics to select groups of tasks to train together.

Using the groups identified by TAG or VTAG, it is possible to define ensembles of MTLs to predict an HR profile where each MTL model is trained independently for a small number of tasks. This is different to the classic MTL approach (MTLaT) of training a single model on all tasks simultaneously. Focusing specifically on TAG or VTAG methodologies to identify groups of tasks that benefit from being trained in parallel (i.e., excluding the use of layer concatenation or PTM for now), our results indicate that using an approach to model design based on first assessing task inter-dependence and then tailoring the model architecture based on the identified inter-dependencies, results in MTL models that consistently outperform STL models. This procedure improves the “average F1-scores over all the output items” and also improves the prediction of the majority of tasks across the three datasets. However, these ensembles of MTLs require a relatively large number of parameters and these models under-perform the classic MTL of all tasks.

The fact that the classic MTL of all tasks outperforms the ensembles of STLs, of TAG- and VTAG-based MTLs shows a beneficial impact of learning all the related tasks in parallel (layers ***h***_3_ and ***h***_4_ for each task, Figure 6B) while using one shared representation (layers ***h***_1_ and ***h***_2_ prior to connecting to the output tasks, Figure 6B). This allows thus the sharing of information to enhance the prediction of all the studied tasks. However, the shared representation aggregates all the information from all the tasks without emphasizing the inter-dependence information between the most related tasks. To address these shortcomings, we kept the idea of tailoring the MTL architecture based on the interdependence between tasks and extended the tailoring approach by using a concatenation method. This results in building up a model of MTL of all tasks, augmented with the concatenation links between the last hidden layer and the output layer for highly inter-dependent tasks (see Section 5.5).

Specifically, we pre-train the MTL of all task architecture and then use the tuned weights of the PTM to initialize the corresponding weights in the concatenated model. The results obtained by these MTL concatenations with pre-trained weights models are consistently higher than the classic MTL of all tasks and the STLs. In general, the performance of both of the concatenation strategies (TAG or VTAG-based) combined with PTM strategies is very similar. Our results indicate that an MTL approach that combines concatenation with PTM improves the prediction of the majority of tasks across the three datasets. Notably, for the two datasets of the IST-3 trial, the prediction of all the tasks by these MTL concatenations with PTM models were all improved indicating that there is no “Robin Hood effect”.

Concatenation is one of the fusion techniques in deep learning that is beneficial for improving the performance of prediction models (64). However, this technique is usually applied to concatenate the information of multiple inputs from different modalities (63, 64) regardless of the inter-dependence between the output tasks. To tackle this limitation, the proposed concatenation between the last hidden layers increases the variation in the input of the output layers and fuses with the inter-dependence between tasks discovered by TAG or VTAG. Therefore, the output layer acquires collective knowledge from all the previous layers and improves efficiency.

Additionally, the performance of MTL of all tasks with concatenation is improved when PTM is applied. PTM is a well-known ML approach that involves re-using the weights of one model to initialize the training of another on the same problem (68). Here, we use the already trained weights of the best-performing MTL of all tasks to initialize the weights of the concatenation model and then continue training the concatenation model, which improves the prediction of all tasks. This fits with the assumption in the literature where PTM leads to positive effects on various artificial intelligence tasks (68).

In summary, all the studied MTLs outperform STLs, regardless of the used grouping strategies, proving the advantage of MTL for improving data efficiency through shared representations and fast learning by leveraging auxiliary information as discussed by Crawshaw (7). In particular, the proposed approach of MTL of all tasks coupled with concatenation strategy and PTM technique has shown to be the most successful model development methodology for this study.

## 8 Conclusion

In conclusion, our results indicate that MTL can outperform STL when predicting patient profiles measured using clinical instruments based on structured questionnaires. The particular use case we have based our analysis on is the BI questionnaire responses for patients between 70 and 120 days post-stroke, and the EQ-5D-3L responses at 6 and 18MaS. For this use case, our results suggest that a modeling approach that combines a pre-trained MTL network for all tasks with a concatenation strategy conditioned by a task grouping method (using TAG or VTAG) improves performance over an STL and a classic MTL baseline when predicting the future health-state of the patient. This work is the first research to predict the individual QoL or ADL items using MTL neural networks.

Our motivation for proposing an MTL approach is that our analysis revealed associations between items in the BI and EQ-5D-3L questionnaires, and MTL enabled us to leverage these associations to improve model performance. More generally, we believe that associations of these types are present in many of the questionnaires used to assess patient profiles (e.g., FIM, SS-QoL, SF-36) and that where these associations exist, MTL is a useful (often overlooked) strategy for the development of clinical decision support systems designed to predict patient outcomes.

Admittedly, the overall average F1-scores of the studied models are not high, which we attribute to the small size of the datasets and the complexity of predicting multiple tasks some with very imbalanced distributions. Consequently, further optimization work is needed to fine-tune the hyper-parameters of the models. Another notable limitation is the exclusion of critical variables such as pathophysiology, pre-morbid conditions, psycho-social status, and access to rehabilitation care in the development of our MTL models. These factors are undeniably central to comprehensively understanding and predicting stroke recovery trajectories. Their exclusion was primarily due to the availability and challenges in quantifying such complex, multifaceted data within the scope of our current dataset. Recognizing that these omissions may affect the model's general ability and depth of prediction, a future direction of our research is to incorporate these dimensions. Integrating a broader range of features will not only enhance the model's performance but also increase its clinical relevance by providing a more holistic view of patient recovery.

For future work, we are actively seeking collaborations and data sources that could enable us to include these critical variables in our analysis, thereby overcoming the current limitations and significantly enriching our predictive capabilities.

## Data Availability

Publicly available datasets were analyzed in this study. The Barthel Index dataset is not readily available because of privacy issues. Requests to access the datasets should be directed to Alejandro García-Rudolph (agarciar@guttmann.com). The IST-3 clinical trial dataset used in this study can be found in the Third International Stroke Trial (IST-3) [http://dx.doi.org/10.7488/ds/1350]. International trials registry ID number: ISRCTN25765518 (https://doi.org/10.1186/ISRCTN25765518).

## Footnotes

^1^This ratio is commonly used for splitting datasets (55) and is suitable in this case because our datasets are relatively small and so we wish to have a large enough training data split to enable the models to train efficiently while still retaining a large enough test sample for evaluation.

^2^Unlike Cramer's V which is a *symmetric* pair-wise affinity measure.
