# Supplementary material for: Multi-task learning for predicting quality-of-life and independence in activities of daily living after stroke: a proof-of-concept study
Source: Front Neurol. 2024 Sep 27;15:1449234. doi: 10.3389/fneur.2024.1449234 (PMC11469734; doi:10.3389/fneur.2024.1449234)
Supplement: Supplementary file 1 [file Data_Sheet_1.PDF]

Supplementary Material

1 ANNEX 1 : BARTHEL INDEX DATASET

The first dataset relating to Barthel Index assessments of patients with stroke during their rehabilitation was collected at the Institute Guttmann (Barcelona, Spain) from 2002 to 2021. Table 2 details the summary statistics of the studied Guttmann dataset. The table shows that there are twice as many males ($n = 130$, 66.7%) as females patients ($n = 65$, 33.3 %) in the dataset. This gender bias is impossible to control in the admitted patients or in the referral from acute treatment units. The mean age of patients is 52.87. There is 137 (70.3%) patients who are married, 38 (19.5%) single, 10 (5.1%) divorced, 8 (4.1%) separated and 2 (1.0%) widowed. Among all these patients with ischemic stroke, 80 (41.0%) patients are with thrombotic stroke, 54 (27.7%) with embolic stroke and 61 (31.3%) with others types. The mean diagnosed NIHSS score of patients is 12.76. On admission, the mean time since injury is 29.59 days after stroke (DaS) and the corresponding mean BI total score of 31.26. At discharge, the mean time since injury is 89.92 DaS and the corresponding mean BI total score of 68.56.

2 ANNEX 2 : EQ-5D-3L AT 6MAS DATASET

The second dataset is extracted from the third international stroke trial (IST-3) dataset Sandercock et al. (2008, 2012) for training models to predict the EQ-5D-3L of patients diagnosed with ischemic stroke at 6-month after stroke (MaS). The demographic, diagnostic information and follow-up information till 6MaS for this dataset are detailed in Table S4.

As shown in Table S4, the mean age is 75.52. There are 352 (48.5%) female and 374 (51.5%) male patients. The mean delay time from stroke to randomisation is 4.02 hours. The mean blood glucose of the considered population is 7.19. Five different stroke subtypes were recorded where 212 (29.2%) patients are with total anterior circulation infarct (TACI), 338 (46.6%) patients with partial anterior circulation infarct (PACI), 106 (14.6%) patients with lacunar infarct (LACI), 68 (9.3%) patients with posterior circulation infarct (POCI) and 2 (0.3%) patients with other stroke subtypes. The mean time from stroke to treatment is 4.32 hours. Adjudicated side of lesion was recorded where 407 (56.1%) patients with the left side, 17 (2.3%) patients with the middle, 301 (41.5%) patients with the right side and 1 (0.1%) unknown. The mean Aspects score for middle cerebral artery is 9 and the mean total Aspect score is 10.99. The mean NIHSS and GCS scores at randomisation is 10.01 and 13.88, respectively.

Follow-up information at 7 DaS shows that the mean number of nights in the Stroke Unit is 4.86 and the mean of total GCS score is 14.84. The mean score for each EQ-5D-3L items at 6MaS is 1.69 for Mobility, 1.63 for Self-care, 1.93 for Usual activities, 1.59 for Pain/Discomfort, 1.58 for Anxiety/Depression and 61.89 for EQ-VAS Health state.

3 ANNEX 3 : EQ-5D-3L AT 18MAS DATASET

The third dataset is also extracted from IST-3 dataset Sandercock et al. (2008, 2012) for training models to predict the EQ-5D-3L of patients diagnosed with ischemic stroke at 18-month after stroke (MaS). Demographic, diagnostic information and follow-up information of this dataset are detailed in Table S6.

As shown in Table S6, the mean age is 74.14. There are 216 (47.8%) female and 236 (52.2%) male patients. The mean delay time from stroke to randomisation is 4 hours. The mean blood glucose of the considered population is 7.1. Five different stroke subtypes was recorded where 125 (27.7%) patients are with TACI, 211 (46.7%) patients with PACI, 75 (16.6%) patients with LACI, 39 (8.6%) patients with POCI and 2 (0.4%) patients with other stroke subtypes. The mean time from stroke to treatment is 4.32 hours. Adjudicated side of lesion was recorded where 264 (58.4%) patients with the left side, 13 (2.9%) patients with the middle, 174 (38.5%) patients with the right side and 1 (0.2%) unknown. The mean Aspects score for middle cerebral artery is 9.03 and the mean total Aspect score is 11.01. The mean NIHSS and GCS scores at randomisation is 9.7 and 13.88, respectively.

Follow-up information at 7 DaS shows that the mean number of nights in the Stroke Unit is 4.74 and the mean of total GCS score is 14.62. Follow-up information at 6MaS shows that the mean OHS score at 6MaS is 2.02. The mean score for each EQ-5D-3L items at 6MaS is 1.8 for Mobility, 1.81 for Self-care, 2.06 for Usual activities, 1.92 for Pain/Discomfort, 1.78 for Anxiety/Depression and 65.28 for EQ-VAS Health state. The mean score for each EQ-5D-3L items at 18MaS is 1.64 for Mobility, 1.57 for Self-care, 1.86 for Usual activities, 1.53 for Pain/Discomfort, 1.51 for Anxiety/Depression and 64.42 for EQ-VAS Health state.

Table S 1. Barthel Index items and scoring schemes

Barthel Index items	Possible Scores	Number of levels
Dressing	[0, 5, 10]	3
Toilet use	[0, 5, 10]	3
Transfers (bed to chair and back)	[0, 5, 10, 15]	4
Stairs	[0, 5, 10]	3
Feeding	[0, 5, 10]	3
Grooming	[0, 5]	2
Mobility on level surfaces	[0, 5, 10, 15]	4
Bladder control	[0, 5, 10]	3
Bowel control	[0, 5, 10]	3
Bathing	[0, 5]	2
Total score	[0, 5, ... 100]	

Table S 2. Demographic, diagnostic and follow-up information of Barthel Index dataset.

		Count	Mode	Mean	STD
Demographics					
Gender		195			
	Male	130			
	Female	65			
Age at Stroke (years)		195	55.30	52.87	9.22
Civil Status		195			
	Married	137			
	Single	38			
	Divorced	10			
	Separated	8			
	Widowed	2			
Diagnostic information					
Ischemic Stroke type		195			
	Thrombotic	80			
	Embolic	54			
	Others	61			
NIHSS		195	15	12.76	5.32

	On admission				At discharge			
**Barthel Index**	**Count**	**Mode**	**Mean**	**STD**	**Count**	**Mode**	**Mean**	**STD**
Time since injury (DaS)	195	34	29.59	9.33	195	71	89.92	14.10
Bathing	195	0	0.36	1.29	195	5	3.21	2.40
Bowel Control	195	10	5.87	4.57	195	10	8.90	2.82
Bladder Control	195	10	5.54	4.61	195	10	8.62	2.94
Mobility	195	0	3.00	4.17	195	15	9.15	5.12
Grooming	195	0	0.97	1.99	195	5	4.23	1.81
Feeding	195	5	4.72	2.10	195	5	6.08	2.12
Stairs	195	0	0.44	1.67	195	0	3.38	3.81
Transfers	195	5	6.31	3.20	195	15	11.54	3.79
Toilet	195	0	2.31	2.74	195	10	7.15	3.36
Dressing	195	0	1.74	2.60	195	5	6.31	3.24
Total	195	10	31.26	20.44	195	90	68.56	23.85

Table S 3. The items in the EQ-5D-3L questionnaire.

Group	Dimensions	Level / Score	Level value
Descriptive dimensions (or items)			
	Mobility		
		I have no problems in walking about	1
		I have some problems in walking about	2
		I am confined to bed	3
	Self-care		
		I have no problems with self-care	1
		I have some problems with washing or dressing myself	2
		I am unable to wash or dress myself	3
	Usual activities		
		I have no problems with performing my usual activities	1
		I have some problems with performing my usual activities	2
		I am unable to perform my usual activities	3
	Pain/discomfort		
		I have no pain or discomfort	1
		I have moderate pain or discomfort	2
		I have extreme pain or discomfort	3
	Anxiety/depression		
		I am not anxious or depressed	1
		I am moderately anxious or depressed	2
		I am extremely anxious or depressed	3
EQ-VAS			
	Overall heath state		
		$[0, 1, \ldots, 100]$	

Table S 4. Demographic, diagnostic and follow-up information of IST-3 dataset for 6MaS modelling study.

Variable name	Count	Mode	Mean	STD
Information at Randomization				
Demographic				
Age	726	82	75.52	12.52
Gender	726			
Female	352			
Male	374			
Clinical/Diagnostic information				
Delay (hours) from stroke to randomisation	726	4.75	4.02	1.21
Blood glucose (mmol/L)	726	6	7.19	2.4
Stroke subtype	726			
Total anterior circulation infarct (TACI)	212			
Partial anterior circulation infarct (PACI)	338			
Lacunar infarct (LACI)	106			
Posterior circulation infarct (POCI)	68			
Other	2			
Time (hours) from stroke to treatment	726	5	4.32	1.19
Adjudicated side of lesion	726			
Left	407			
Middle	17			
Right	301			
Unknown	1			
Aspects score (max 10) for Middle cerebral artery	726	10	9	2
Total aspects score (max 12)	726	12	10.99	2.01
NIHSS	726	5	10.01	6.4
Glasgow Coma Scale				
Best eye response	726	4	3.86	0.45
Best motor response	726	6	5.85	0.57
Best verbal response	726	5	4.18	1.28
Total score	726	15	13.88	1.77
Follow-up information				
at 7 Days after Stroke				
Number of nights in Stroke Unit	726	7	4.86	2.3
Glasgow Coma Scale				
Best eye response	726	4	4.05	1.37
Best motor response	726	6	6.04	1.23
Best verbal response	726	5	4.75	1.54
Total score	726	15	14.84	3.98
at 6 Months after Stroke				
EQ-5D-3L (EuroQoL)				
Mobility	726	2	1.69	0.64
Self-Care	726	1	1.63	0.77
Usual activities	726	2	1.93	0.78
Pain / Discomfort	726	1	1.59	0.62
Anxiety / Depression	726	1	1.58	0.65
EQ-VAS Health state	726	50	61.89	22.75
Binned EQ-VAS Health state	726	[51,75]		

Table S 5. Input and output variables of ITS-3 dataset for EQ-5D-3L 6MaS modelling.

	Variable name	Count	Cardinality	Mode	Mean	STD	Variable label
Input							
	age	726	57	82	75.52	12.52	Age at randomisation
	gender	726	2	Male			Gender
	randdelay	726	218	4.75	4.02	1.21	Delay (hours) from stroke to randomisation
	livealone_rand	726	2	0			Lived alone before stroke? (Yes/No)
	infarct	726	3	0			Recent ischaemic change likely cause of this stroke?
	antiplat_rand	726	2	0			Received antiplatelet drugs in last 48 hours? (Yes/No)
	atrialfib_rand	726	2	0			Patient in atrial fibrillation at randomisation? (Yes/No)
	sbprand	726	106	160	154.95	22.86	Systolic BP at randomisation (mm Hg)
	dbprand	726	68	80	81.91	13.9	Diastolic BP at randomisation (mm Hg)
	weight	726	77	70	73.13	14.7	Estimated weight (kg)
	glucose	726	16	6	7.19	2.4	Blood glucose (mmol/L)
	gcs_eye_rand	726	4	4	3.86	0.45	Best eye response (Glasgow Coma Scale) at randomisation
	gcs_motor_rand	726	6	6	5.85	0.57	Best motor response (Glasgow Coma Scale) at randomisation
	gcs_verbal_rand	726	5	5	4.18	1.28	Best verbal response (Glasgow Coma Scale) at randomisation
	gcs_score_rand	726	11	15	13.88	1.77	Total Glasgow Coma Scale score at randomisation
	nihss	726	32	5	10.01	6.4	Total NIH Stroke Score at randomisation
	liftarms_rand	726	2	1			Able to lift both arms off bed at randomisation (Yes/No)
	ablewalk_rand	726	2	0			Able to walk without help at randomisation (Yes/No)
	weakface_rand	726	3	1			Unilateral weakness affecting face at randomisation
	weakarm_rand	726	3	1			Unilateral weakness affecting arm or hand at randomisation
	weakleg_rand	726	3	1			Unilateral weakness affecting leg or foot at randomisation
	dysphasia_rand	726	3	2			Dysphasia at randomisation
	hemianopia_rand	726	3	2			Homonymous hemianopia at randomisation
	visuospat_rand	726	3	2			Visuospatial disorder at randomisation
	brainstemsigns_rand	726	3	2			Brainstem or cerebellar signs at randomisation
	otherdeficit_rand	726	3	2			Other neurological deficit at randomisation
	stroketype	726	5	PACI			Stroke subtype
	treatdelay	726	201	5	4.32	1.19	Time (hr) from stroke to treatment
	asl	726	4	Left			Adjudicated side of lesion
	aspirin_day1	726	2	0			Aspirin in first 24 hours? (Yes/No)
	antiplatelet_day1	726	2	0			Other antiplatelets in first 24 hours? (Yes/No)
	lowdose_heparin_day1	726	2	0			Low dose heparin or low molecular weight heparin in first 24 hours? (Yes/No)
	full_anticoag_day1	726	2	0			Full anti-coagulation in first 24 hours? (Yes/No)
	lowerBP_day1	726	2	0			Treatment to lower blood pressure in first 24 hours? (Yes/No)
	iv_fluids_day1	726	4	1			Intravenous fluids in first 24 hours?
	insulin_day1	726	3	2			Insulin in first 24 hours?
	aspirin_days2to7	726	3	1			Aspirin between 24 hours & 7 days?
	antiplatelet_days2to7	726	2	0			Other antiplatelets between 24 hours & 7 days? (Yes/No)
	lowdose_heparin_days2to7	726	3	2			Low dose heparin or low molecular weight heparin between 24 hours & 7 days?

	Variable name	Count	Cardinality	Mode	Mean	STD	Variable label
	full_anticoag_days2to7	726	2	0			Full anti-coagulation between 24 hours & 7 days? (Yes/No)
	lowerBP_days2to7	726	2	1			Treatment to lower blood pressure between 24 hours & 7 days? (Yes/No)
	nasogastric_days2to7	726	3	2			Nasogastric tube or percutaneous gastrostomy between 24 hours & 7 days?
	antibiotics_days2to7	726	3	2			Antibiotics between 24 hours & 7 days?
	brainsite7	726	3	1			Location of initial ischaemic stroke (7-day form)
	med_adno	726	7	0	0.04	0.41	Number of nights in Medical Admissions Unit in first 7 days
	critcareno	726	8	0	0.4	1	Number of nights in Critical Care Unit in first 7 days
	strk_unitno	726	8	7	4.86	2.3	Number of nights in Stroke Unit in first 7 days
	genwardno	726	8	0	0.66	1.77	Number of nights in General Ward in first 7 days
	adjudicated	726	2	0			Patient had adjudicated event in first 7 days (Yes/No)
	sevendaycase	726	5	0			Type of adjudicated event in first 7 days
	gcs_eye_7	726	5	4	4.05	1.37	Best eye response at 7 days (Glasgow Coma Scale)
	gcs_motor_7	726	6	6	6.04	1.23	Best motor response at 7 days (Glasgow Coma Scale)
	gcs_verbal_7	726	6	5	4.75	1.54	Best verbal response at 7 days (Glasgow Coma Scale)
	liftarms_7	726	3	1	1.3	1.27	Able to lift both arms off bed at 7 days
	ablewalk_7	726	3	1	1.48	1.29	Able to walk without help at 7 days
	indepinadl_7	726	3	1	1.53	1.29	Independent in activities of daily living at 7 days
	R_infarct_size	726	5	0			Infarct size (from R scan)
	R_infarct_territory	726	4	0			Infarct territory (from R scan)
	R_hypodensity	726	3	0			Degree of acute hypodensity (R scan)
	R_swelling	726	3	0			Degree of tissue swelling in acute infarct (R scan)
	R_hyperdense_arteries	726	3	0			Hyperdense arteries visible on R scan
	R_mca_aspects	726	11	10	9	2	Aspects score (max 10) for Middle cerebral artery (R scan)
	R_tot_aspects	726	11	12	10.99	2.01	Total aspects score (max 12) including ACA/PCA (R scan)
	vis_infarct	726	2	0			Visible signs of infarct (R scan) (Yes/No)
Output							
	mobility6	726	3	2	1.69	0.64	EQ-5D Mobility problems (6-month form)
	selfcare6	726	3	1	1.63	0.77	EQ-5D Washing or dressing problems (6-month form)
	activities6	726	3	2	1.93	0.78	EQ-5D Usual activities problems (6-month form)
	pain6	726	3	1	1.59	0.62	EQ-5D Pain or discomfort (6-month form)
	anxiety6	726	3	1	1.58	0.65	EQ-5D Anxiety or depression (6-month form)
	euroqol6	726	72	50	61.89	22.75	EQ-VAS Health state (Euroqol) at 6 months
	euroqol6bin	726	4	[51,75]			Binning health state (Euroqol) at 6 months

Table S 6. Demographic, diagnostic and follow-up information of IST-3 dataset for 18MaS modelling study.

Variable name	Count	Mode	Mean	STD
Information at Randomization				
Demographic				
Age	452	82	74.14	12.93
Gender	452			
Female	216			
Male	236			
Clinical/Diagnostic information				
Delay (hours) from stroke to randomisation	452	3.5	4	1.19
Blood glucose (mmol/L)	452	6	7.1	2.45
Stroke subtype	452			
Total anterior circulation infarct (TACI)	125			
Partial anterior circulation infarct (PACI)	211			
Lacunar infarct (LACI)	75			
Posterior circulation infarct (POCI)	39			
Other	2			
Time (hours) from stroke to treatment	452	5.92	4.32	1.17
Adjudicated side of lesion	452			
Left	264			
Middle	13			
Right	174			
Unknown	1			
Aspects score for Middle cerebral artery	452	10	9.03	1.89
Total aspects score	452	12	11.01	1.88
NIHSS	452	5	9.7	6.39
Glasgow Coma Scale				
Best eye response	452	4	3.87	0.41
Best motor response	452	6	5.84	0.62
Best verbal response	452	5	4.17	1.31
Total score	452	15	13.88	1.78
Follow-up information				
at 7 Days after Stroke				
Number of nights in Stroke Unit	452	7	4.74	2.36
Glasgow Coma Scale				
Best eye response	452	4	3.96	0.28
Best motor response	452	6	5.95	0.43
Best verbal response	452	5	4.71	0.81
Total score	452	15	14.62	1.28
at 6 Months after Stroke				
Oxford Handicap Score	452	1	2.02	1.48
EQ-5D-3L (EuroQoL)				
Mobility	452	2	1.8	2.02
Self-Care	452	1	1.81	2.39
Usual activities	452	2	2.06	2.21
Pain / Discomfort	452	1	1.92	2.64
Anxiety / Depression	452	1	1.78	2.2
EQ-VAS Health state	452	80	65.28	21.01
at 18 Months after Stroke				
EQ-5D-3L (EuroQoL)				
Mobility	452	2	1.64	0.61
Self-Care	452	1	1.57	0.74
Usual activities	452	2	1.86	0.77
Pain / Discomfort	452	1	1.53	0.58
Anxiety / Depression	452	1	1.51	0.6
EQ-VAS Health state	452	70	64.42	22.24
Binned EQ-VAS Health state	452	[51,75]		

Table S 7. Input and output variables of ITS-3 dataset for EQ-5D-3L 18MaS modelling.

	Variable name	Count	Cardinality	Mode	Mean	STD	
Input							
	age	452	54	82	74.14	12.93	Age at randomisation
	gender	452	2	Male	1.52	0.5	Gender
	randdelay	452	174	3.5	4	1.19	Delay (hours) from stroke to randomisation
	livealone_rand	452	2	2			Lived alone before stroke? (Yes/No)
	infarct	452	3	0			Recent ischaemic change likely cause of this stroke?
	antiplat_rand	452	2	2			Received antiplatelet drugs in last 48 hours? (Yes/No)
	atrialfib_rand	452	2	2			Patient in atrial fibrillation at randomisation? (Yes/No)
	sbprand	452	97	130	155.11	22.94	Systolic BP at randomisation (mm Hg)
	dbprand	452	66	80	82.79	13.55	Diastolic BP at randomisation (mm Hg)
	weight	452	70	70	73.97	14.34	Estimated weight (kg)
	glucose	452	16	6	7.1	2.45	Blood glucose (mmol/L)
	gcs_eye_rand	452	4	4	3.87	0.41	Best eye response (Glasgow Coma Scale) at randomisation
	gcs_motor_rand	452	6	6	5.84	0.62	Best motor response (Glasgow Coma Scale) at randomisation
	gcs_verbal_rand	452	5	5	4.17	1.31	Best verbal response (Glasgow Coma Scale) at randomisation
	gcs_score_rand	452	11	15	13.88	1.78	Total Glasgow Coma Scale score at randomisation
	nihss	452	29	5	9.7	6.39	Total NIH Stroke Score at randomisation
	liftarms_rand	452	2	1			Able to lift both arms off bed at randomisation (Yes/No)
	ablewalk_rand	452	2	2			Able to walk without help at randomisation (Yes/No)
	weakface_rand	452	3	1			Unilateral weakness affecting face at randomisation
	weakarm_rand	452	3	1			Unilateral weakness affecting arm or hand at randomisation
	weakleg_rand	452	3	1			Unilateral weakness affecting leg or foot at randomisation
	dysphasia_rand	452	3	2			Dysphasia at randomisation
	hemianopia_rand	452	3	2			Homonymous hemianopia at randomisation
	visuospat_rand	452	3	2			Visuospatial disorder at randomisation
	brainstemsigns_rand	452	3	2			Brainstem or cerebellar signs at randomisation
	otherdeficit_rand	452	3	2			Other neurological deficit at randomisation
	stroketype	452	5	PACI			Stroke subtype
	treatdelay	452	163	5.92	4.32	1.17	Time (hr) from stroke to treatment
	asl	452	4	Left			Adjudicated side of lesion
	aspirin_day1	452	2	2			Aspirin in first 24 hours? (Yes/No)
	antiplatelet_day1	452	2	2			Other antiplatelets in first 24 hours? (Yes/No)
	lowdose_heparin_day1	452	2	2			Low dose heparin or low molecular weight heparin in first 24 hours? (Yes/No)

	Variable name	Count	Cardinality	Mode	Mean	STD	
Input							
	full_anticoag_day1	452	2	2			Full anti-coagulation in first 24 hours? (Yes/No)
	lowerBP_day1	452	2	2			Treatment to lower blood pressure in first 24 hours? (Yes/No)
	iv_fluids_day1	452	4	1			Intravenous fluids in first 24 hours?
	insulin_day1	452	3	2			Insulin in first 24 hours?
	aspirin_days2to7	452	3	1			Aspirin between 24 hours & 7 days?
	antiplatelet_days2to7	452	2	2			Other antiplatelets between 24 hours & 7 days? (Yes/No)
	lowdose_heparin_days2to7	452	3	2			Low dose heparin or low molecular weight heparin between 24 hours & 7 days?
	full_anticoag_days2to7	452	2	2			Full anti-coagulation between 24 hours & 7 days? (Yes/No)
	lowerBP_days2to7	452	2	1			Treatment to lower blood pressure between 24 hours & 7 days? (Yes/No)
	nasogastric_days2to7	452	3	2			Nasogastric tube or percutaneous gastrostomy between 24 hours & 7 days?
	antibiotics_days2to7	452	3	2			Antibiotics between 24 hours & 7 days?
	brainsite7	452	3	1			Location of initial ischaemic stroke (7 day form)
	med_adno	452	6	0	0.05	0.46	Number of nights in Medical Admissions Unit in first 7 days
	critcareno	452	7	0	0.5	1.09	Number of nights in Critical Care Unit in first 7 days
	strk_unitno	452	8	7	4.74	2.36	Number of nights in Stroke Unit in first 7 days
	genwardno	452	8	0	0.78	1.91	Number of nights in General Ward in first 7 days
	adjudicated	452	2	0			Patient had adjudicated event in first 7 days (Yes/No)
	sevendaycase	452	5	0			Type of adjudicated event in first 7 days
	gcs_eye_7	452	4	4	3.96	0.28	Best eye response at 7 days (Glasgow Coma Scale)
	gcs_motor_7	452	4	6	5.95	0.43	Best motor response at 7 days (Glasgow Coma Scale)
	gcs_verbal_7	452	5	5	4.71	0.81	Best verbal response at 7 days (Glasgow Coma Scale)
	liftarms_7	452	2	1	1.17	0.38	Able to lift both arms off bed at 7 days
	ablewalk_7	452	2	1	1.35	0.48	Able to walk without help at 7 days
	indepinadl_7	452	2	1	1.4	0.49	Independent in activities of daily living at 7 days
	ohs6	452	6	1	2.02	1.48	OHS from 6-month form
	aspirin6	452	4	3			Aspirin given on admission day (6-month form)
	bloodthin6	452	4	3			Blood thinning injections given on admission day (6-month form)
	clotbust6	452	4	1			Clot busting drugs given on admission day (6-month form)
	stocking6	452	4	2			Special stockings given on admission day (6-month form)
	gotprobs6	452	3	1			Stroke left patient with problems (6-month form)
	needhelp6	452	3	2			Needs help with everyday activities (6-month form)
	walkhelp6	452	3	2			Needs help to walk (6-month form)
	speakprob6	452	3	2			Major problems speaking (6-month form)
	mobility6	452	4	2	1.8	2.02	EQ-5D Mobility problems (6-month form)
	selfcare6	452	4	1	1.81	2.39	EQ-5D Washing or dressing problems (6-month form)
	activities6	452	4	2	2.06	2.21	EQ-5D Usual activities problems (6-month form)
	pain6	452	4	1	1.92	2.64	EQ-5DPain or discomfort (6-month form)
	anxiety6	452	4	1	1.78	2.2	EQ-5D Anxiety or depression (6-month form)
	wherelive6	452	4	1			Where patient lives now (6-month form, patient not in hospital)
	howlive6	452	3	2			How patient lives now (6-month form, patient not in hospital)
	euroqol6	452	65	80	65.28	21.01	EQ-VAS Health state (Euroqol) at 6 months

	Variable name	Count	Cardinality	Mode	Mean	STD	
Input							
	R_infarct_size	452	5	0	0.74	1.18	Infarct size (from R scan)
	R_infarct_territory	452	4	0	0.36	0.55	Infarct territory (from R scan)
	R_hypodensity	452	3	0	0.38	0.59	Degree of acute hypodensity (R scan)
	R_swelling	452	3	0	0.18	0.43	Degree of tissue swelling in acute infarct (R scan)
	R_hyperdense_arteries	452	3	0	0.18	0.42	Hyperdense arteries visible on R scan
	R_mca_aspects	452	11	10	9.03	1.89	Aspects score (max 10) for Middle cerebral artery (R scan)
	R_tot_aspects	452	11	12	11.01	1.88	Total aspects score (max 12) including ACA/PCA (R scan)
	vis_infarct	452	2	0			Visible signs of infarct (R scan) (Yes/No)
Output							
	mobility18	452	3	2	1.64	0.61	EQ-5D Mobility problems (18-month form)
	selfcare18	452	3	1	1.57	0.74	EQ-5D Washing or dressing problems (18-month form)
	activities18	452	3	2	1.86	0.77	EQ-5D Usual activities problems (18-month form)
	pain18	452	3	1	1.53	0.58	EQ-5D Pain or discomfort (18-month form)
	anxiety18	452	3	1	1.51	0.6	EQ-5D Anxiety or depression (18-month form)
	euroqol18	452	55	70	64.42	22.24	EQ-VAS Health state (Euroqol) at 18 months
	euroqol18bin	452	4	[51, 75]			Binning EQ-VAS health state (Euroqol) at 18 months

Table S 8. Average, Min, Max and Standard deviation (STD) of the number of trainable parameter of studied models : ensemble of STLs, TAG-based MTLs, VTAG-based MTLs, classic MTL of all tasks, MTL of all tasks with concatenation conditioned by TAG and VTAG with and without pre-trained model. Results are averaged over 50 trained architectures on 50 Monte Carlo data splits.

Dataset	Model	Average	Min	Max	STD
BI					
	eSTL	348 828	348 828	348 828	0
	eTAG-MTL	355 498.5	332 032	442 844	31 814.06
	eVTaG-MTL	439 261.1	387 337	442 945	13 228.08
	MTLaT	219 228	219 228	219 228	0
	MTLaT-ccTAG	222 028	222 028	222 028	0
	MTLaT-ccTAG-PTM	222 028	222 028	222 028	0
	MTLaT-ccVTaG	222 028	222 028	222 028	0
	MTLaT-ccVTaG-PTM	222 028	222 028	222 028	0
EQ-5D-3L 6MaS					
	eSTL	272 519	272 519	272 519	0
	eTAG-MTL	326 994.8	263 725	329 631	13 046
	eVTaG-MTL	328 438.1	263 725	329 833	9338.718
	MTLaT	148 019	148 019	148 019	0
	MTLaT-ccTAG	149 919	149 919	149 919	0
	MTLaT-ccTAG-PTM	149 919	149 919	149 919	0
	MTLaT-ccVTaG	149 919	149 919	149 919	0
	MTLaT-ccVTaG-PTM	149 919	149 919	149 919	0
EQ-5D-3L 18MaS					
	eSTL	295 919	295 919	295 919	0
	eTAG-MTL	336 565.9	279 325	349 131	27 090.87
	eVTaG-MTL	347 934.9	279 426	349 333	9886.343
	MTLaT	151 919	151 919	151 919	0
	MTLaT-ccTAG	153 819	153 819	153 819	0
	MTLaT-ccTAG-PTM	153 819	153 819	153 819	0
	MTLaT-ccVTaG	153 819	153 819	153 819	0
	MTLaT-ccVTaG-PTM	153 819	153 819	153 819	0

REFERENCES

Sandercock P, Lindley R, Wardlaw J, Dennis M, Lewis S, Venables G, et al. The third international stroke trial (IST-3) of thrombolysis for acute ischaemic stroke. *Trials* **9** (2008) 37. doi:10.1186/1745-6215-9-37.

Sandercock P, Wardlaw JM, Lindley RI, Dennis M, Cohen G, Murray G, et al. The benefits and harms of intravenous thrombolysis with recombinant tissue plasminogen activator within 6 h of acute ischaemic stroke (the third international stroke trial [IST-3]): A randomised controlled trial. *The Lancet* **379** (2012) 2352–2363. doi:10.1016/S0140-6736(12)60768-5.
